# Regulating kinetics and thermodynamics of electrochemical nitrogen reduction with metal single-atom catalysts in a pressurized electrolyser

**DOI:** 10.1073/pnas.2015108117

**Published:** 2020-11-10

**Authors:** Haiyuan Zou, Weifeng Rong, Shuting Wei, Yongfei Ji, Lele Duan

**Affiliations:** ^a^Department of Chemistry, Southern University of Science and Technology, 518055 Shenzhen, Guangdong, China;; ^b^School of Chemistry and Chemical Engineering, Harbin Institute of Technology, 150001 Harbin, China;; ^c^School of Chemistry and Chemical Engineering, Guangzhou University, 510006 Guangzhou, China;; ^d^Shenzhen Grubbs Institute, Southern University of Science and Technology, 518055 Shenzhen, Guangdong, China

**Keywords:** single-atom catalysts, graphdiyne, pressurized electrocatalysis, nitrogen reduction reaction

## Abstract

Using renewable electricity to synthesize ammonia from nitrogen paves a sustainable route to making value-added chemicals but yet requires further advances in electrocatalyst development and device integration. By engineering both electrocatalyst and electrolyzer to simultaneously regulate chemical kinetics and thermodynamic driving forces of the electrocatalytic nitrogen reduction reaction (ENRR), we report herein stereoconfinement-induced densely populated metal single atoms (Rh, Ru, Co) on graphdiyne (GDY) matrix (formulated as M SA/GDY) and realized a boosted ENRR activity in a pressurized reaction system. Remarkably, under the pressurized environment, the hydrogen evolution reaction of M SA/GDY was effectively suppressed and the desired ENRR activity was strongly amplificated. As a result, the pressurized ENRR activity of Rh SA/GDY at 55 atm exhibited a record-high NH_3_ formation rate of 74.15 μg h^−1^⋅cm^−2^, a Faraday efficiency of 20.36%, and a NH_3_ partial current of 0.35 mA cm^−2^ at −0.20 V versus reversible hydrogen electrode, which, respectively, displayed 7.3-, 4.9-, and 9.2-fold enhancements compared with those obtained under ambient conditions. Furthermore, a time-independent ammonia yield rate using purified ^15^N_2_ confirmed the concrete ammonia electroproduction. Theoretical calculations reveal that the driving force for the formation of end-on N_2_* on Rh SA/GDY increased by 9.62 kJ/mol under the pressurized conditions, facilitating the ENRR process. We envisage that the cooperative regulations of catalysts and electrochemical devices open up the possibilities for industrially viable electrochemical ammonia production.

## Significance

The present-day industrial ammonia synthesis is overreliance on the Haber–Bosch process, yet consumes more than 1% of the global energy supply along with gigatonne greenhouse-gas emission per year. Electrochemical nitrogen reduction reaction (ENRR) offers a sustainable path to produce ammonia under mild conditions, while its efficiency achieved by far is fairly low, which requires the development of both catalysts and electrolyzers. Here, by the cooperation of densely populated metal single atoms on graphdiyne substrate with the pressurized electrocatalytic system, the kinetics and the thermodynamic driving force of ENRR are effectively regulated, leading to a record-high ammonia yield rate. This work motivates the technological and material coevolution for the ENRR toward its envisioned application.

Ammonia is essential for human propagation and thriving (1, 2). Today’s global ammonia production is excessively dependent on the Haber–Bosch method, which converts nitrogen and hydrogen to ammonia at high temperature (300–500 °C) and pressure (200–300 atm) (3). So far, this century-old strategy has contributed vastly annual productions, yet significantly exacerbating the global energy consumption and greenhouse-gas emission. Electrocatalytic N_2_ reduction reaction (ENRR) to synthesize ammonia from nitrogen and water under mild conditions represents a viable alternative that strategically transforms the energy-intensive sector toward sustainability, while its efficiency achieved so far is fairly low (4–8).

The primary hurdle obstructing the ENRR lies in issues such as the inherent inertness of N_2_, the high-energy barrier of N_2_ activation, multiple electron–proton transfers, the low solubility of N_2_ in aqueous solutions and competing hydrogen evolution reaction (HER), etc. (9–12). On the basis of these premises, strategies are highlighted to modulate the kinetics and thermodynamic equilibrium of the progress, thus steering the reaction toward the production of ammonia while mitigating HER (13–16). From a kinetic perspective, many catalyst-centric approaches, such as introducing alloy, defects, doping, and strain, etc., have been explored to improve nitrogen reduction performance (17–21). The overall ENRR efficiency, however, is still insufficient to meet the practical requirements. On the other hand, the improvement of the thermodynamic driving force for ammonia production, such as regulating electrochemical reaction conditions, may offer equally positive effects to efficiently promote the N_2_ reduction process and suppress the unwanted side reactions (22). Conventionally, exploration of the innovation of electrocatalysts or electrochemical cell devices has always been undergone independently, despite their indivisible interconnection nature. Indeed, the ENRR advancements toward the envisioned practical applications depend very much on the cooperative development of both electrocatalysts and electrochemical cell devices (23).

Given that the reductive N_2_ adsorption (N_2_ + e^−^ + H^+^ → *N = NH) is usually regarded as the potential limiting step, novel metal single-atom catalysts (SACs, e.g., Ru, Rh, Co) with a favorable ENRR kinetics guarantee a great promise to circumvent the N_2_ activation energy barrier (24–27). These catalysts, on the other hand, also suffer vigorous competition from HER and low content of metal loading (28–30). Encouragingly, the most recent research work demonstrated that the system-level regulation of the pressurized electrocatalytic environment could affect the chemical equilibrium of the ammonia production reaction and meanwhile endow tangible HER suppression (31). It thus warrants research efforts to query whether the integration of SACs with pressurized electrochemical environments will lever synergies between kinetics and thermodynamic driving forces and be the game-changer for the ENRR.

Herein, we showcase that SACs-catalyzed N_2_ reduction in a pressurized system is an effective design principle to enhance both the chemical kinetics and thermodynamic process, leading to the amplified ENRR activity with simultaneously retarded HER. The deployed SACs contain Ru, Rh, and Co atoms featured with densely populated active sites and stabilized on graphdiyne (GDY) support (referred to as M SA/GDY; M = Rh, Ru, and Co); these electrocatalysts were prepared by a facile and mild method via stereoconfinement of metal atoms on the GDY framework. Through extensive ENRR test using adequately cleaned N_2_, we found that the as-prepared M SA/GDY electrocatalysts render prominently enhanced ammonia electroproduction with obvious HER inhibition at the pressurized electrocatalytic system, suggesting positive cooperation between SAC and the pressurized environment. Remarkably, a record-high ammonia yield rate of 74.15 μg h^−1^⋅cm^−2^, a Faraday efficiency (FE) of 20.36%, and a NH_3_ partial current density of 0.35 mA cm^−2^ were achieved for Rh SA/GDY at 55 atm of N_2_, which shows 7.3-, 4.9-, and 9.2-fold enhancement in comparison with those obtained in ambient conditions, outperforming the state-of-art ENRR catalysts. Additionally, a time-independent ammonia yield rate using adequately cleaned ^15^N_2_ ensured the ammonia electrosynthesis from N_2_.

## Results

### Synthesis and Characterizations.

**Fig. 1.**
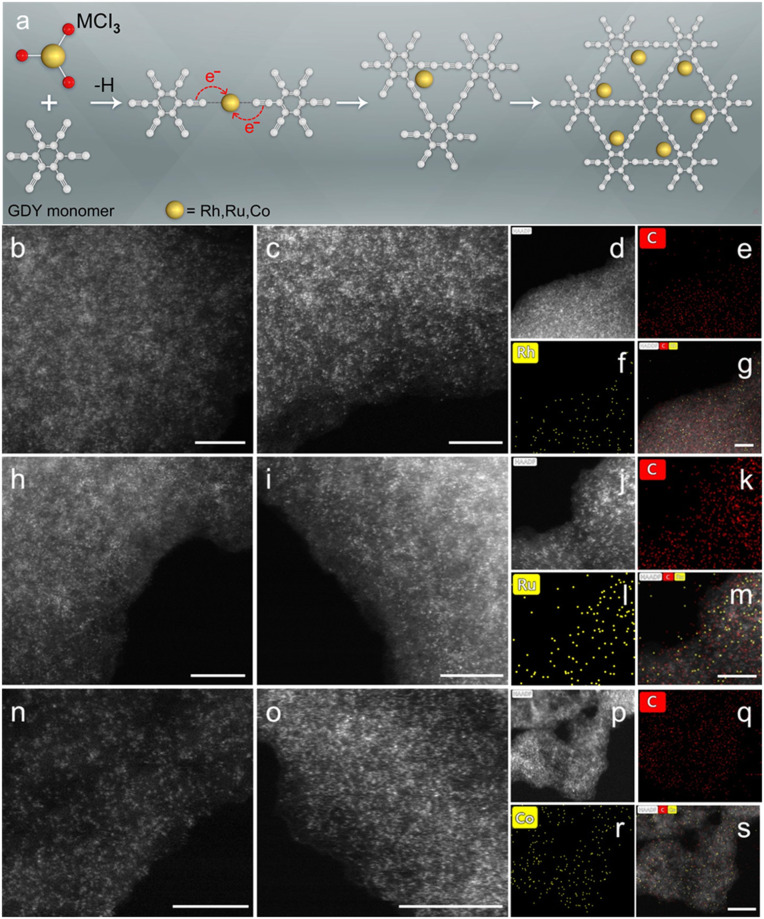
(*A*) Schematic illustration of the fabrication of M SA/GDY (M = Rh, Ru, and Co). (*B*, *C*, *H*, *I*, *N,* and *O*) HAADF-STEM images of Rh, Ru, and Co SA/GDY. (*D*–*G*, *J*–*M*, and *P*–*S*) Corresponding EDX mapping of M SA/GDY (Scale bar, 5 nm.)

The synthetic rationale of M SA/GDY preparation was based on the well-established reductive elimination reactions for the C−C coupling, such as Suzuki, Negishi, and Sonogashira cross-coupling progress (32–34). Typically, for the reductive elimination step, substrates and a metal cation form coordination complexes, and then the metal center accepts electrons from the substrates, leading to the formation of C−C bond and the low-valent metal center. We adopted this reaction mechanism and designed an efficient, one-pot synthetic procedure (Fig. 1*A*) for the preparation of high-loading M SA/GDY (M = Rh, Ru, and Co) materials. Metal trichloride of RhCl_3_, RuCl_3,_ and CoCl_3_ were used as metal precursors to efficiently drive the cross-coupling process thanks to their strong oxidation ability and suitable solubility in pyridine. In the presence of a base (pyridine), the GDY monomer can be deprotonated to a certain degree and form a coordination complex with Rh^3+^, Ru^3+^, and Co^3+^; then, the cross-coupling of GDY monomers occurs at elevated temperature, resulting in the formation of GDY with reduced metal atoms. The reduced metal atoms were singly immobilized on the GDY framework through stereoconfinement, yielding the desired products. Significantly, our synthetic process was proceeded by one pot under a mild temperature of 60 °C, which dramatically decreases the energy consumption (*SI Appendix*, *Experiment section*). The obtained M SA/GDY samples were primarily subjected to a close structure and morphology analysis. Raman spectra of these samples show three different bands, ascribing to the D band (1,365 cm^−1^), G band (1,534 cm^−1^), and conjugated diyne linkage vibration (2,167 cm^−1^) (*SI Appendix*, Fig. S1*A*) (35–37). Their X-ray diffraction patterns showed a sole broad feature at ∼22° corresponding to the interlayer distance of the carbon matrix and excluding the absence of any crystalline species in the M SA/GDY (*SI Appendix*, Fig. S1*B*) (38). Further, the scanning transmission electron microscopy (STEM) images recorded with aberration-corrected high-angle annular dark-field (HAADF) detector at various regions evidenced the atomic-level dispersion of highly concentrated metal atoms (Rh, Ru, Co) by Z-contrast analysis (Fig. 1 *B*, *C*, *H*, *I*, *N*, and *O* and *SI Appendix*, Fig. S2). Moreover, the atomic-scale energy-dispersive X-ray spectroscopy mapping revealed the distinguishable element signals of C and isolated metal atoms (Rh, Ru, Co) across uniformly in the GDY matrix (Fig. 1 *D*–*G*, *J*–*M*, and *P*–*S*). Inductively coupled plasma optical emission spectrometry analysis confirmed the weight fraction of corresponding metals to be 13.22, 15.31, and 12.08 wt % for Rh, Ru, and Co, respectively. In principle, this simple one-pot synthetic method can be generally extended to prepare other metal single-atom catalysts by exchanging the metal precursors with proper oxidation power.

**Fig. 2.**
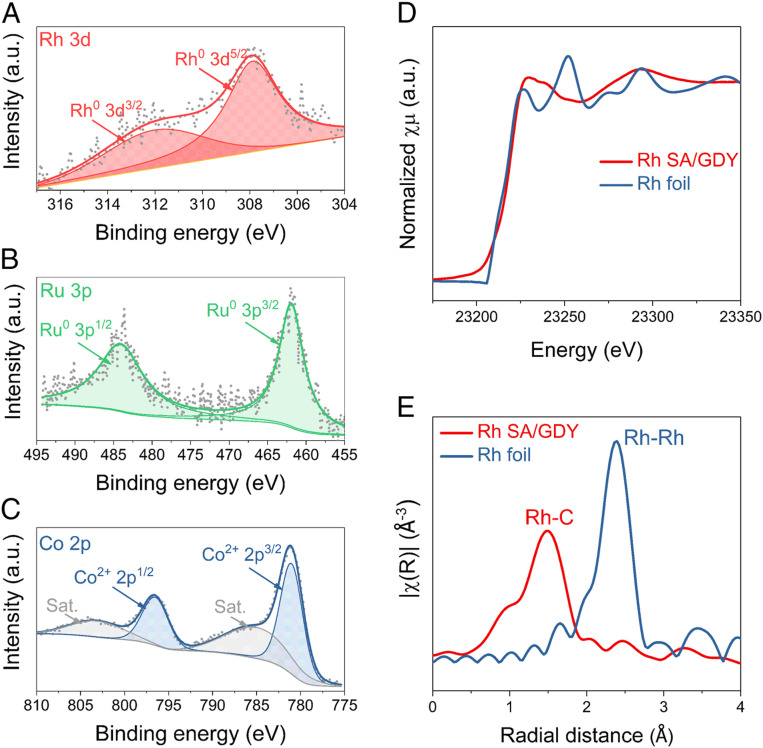
XPS and XAS spectra of M SA/GDY. (*A*–*C*) Deconvoluted Rh 3*d*, Ru 3*p,* and Co 2*p* XPS spectra. (*D*) Rh K-edge XANES and (*E*) FT-EXAFS spectra of Rh SA/GDY, along with reference Rh foils.

To uncover the chemical states and atomic coordination environment of M SA/GDY, X-ray photoelectron spectroscopy (XPS) and synchrotron-based X-ray absorption spectroscopy (XAS) were performed. As shown in *SI Appendix*, Fig. S3, representative XPS survey spectra show two major characteristic peaks of C 1*s* and O 1*s* at 284.6 and 532 eV, respectively. The core-level XPS spectra of Rh 3*d* and Ru 3*p* can be deconvoluted into spin-orbit peaks, corresponding to the Rh^0^ at around 307.5 and 312.2 eV, Ru^0^ at 461.8 and 483.7 eV (Fig. 2 *A* and *B*) (39, 40). The reduced valence state of the metal center suggests that the reductive elimination process indeed occurs as expected, providing the driving force for the GDY formation and metal deposition. It is important to point out that the high-resolution Co 2*p* profile mainly comprises Co^2+^ at around 780.5 and 796.4 eV, which arises from the easy oxidation of Co atoms (Fig. 2*C*) (41). Furthermore, the X-ray absorption near-edge spectroscopy (XANES, Fig. 2*D*) revealed that the absorption edge of Rh SA/GDY is overlapped well with that of Rh foil, in agreement with the zero valence feature of the Rh single atoms. The Fourier-transformed extended X-ray absorption fine structure (FT-EXAFS) was applied to explore the precise coordination structure. As shown in Fig. 2*E*, a sole and dominant peak at ∼1.5 Å is ascribed to the Rh−C bond, which is shorter than the Rh−Rh bond at 2.4 Å, suggesting the salient single-atom feature. In addition, EXAFS fitting shows that the Rh coordination number is 3.92, close to 4, demonstrating the presence of Rh atoms coordinated by four surrounding carbon atoms (*SI Appendix*, Fig. S4 and Table S1).

### ENRR Tests.

**Fig. 3.**
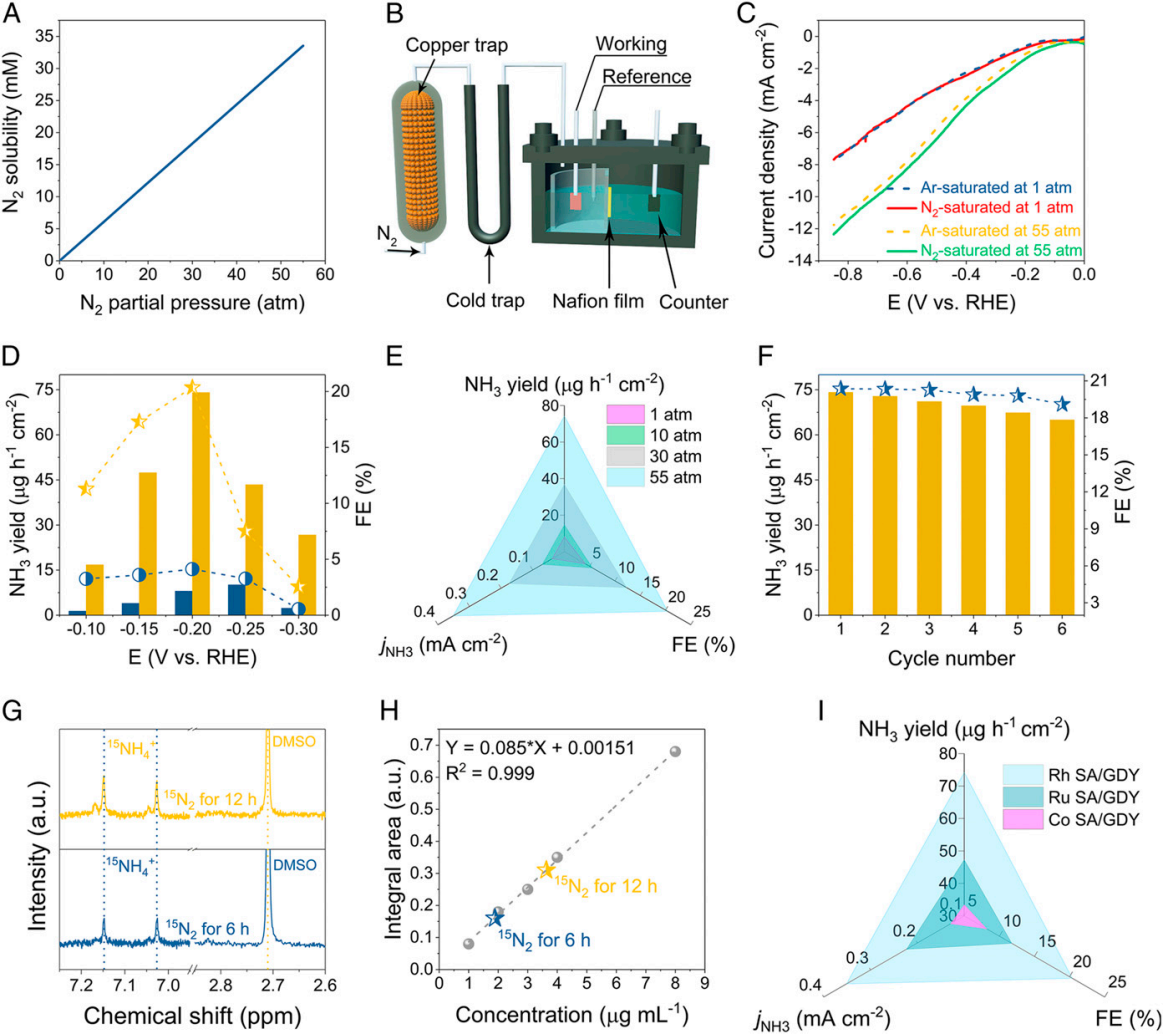
Pressurized ENRR measurements. (*A*) A plot of the solubility of N_2_ gas versus the partial pressure. (*B*) Schematic of the home-made pressurized ENRR setup. (*C*) Ar- and N_2_-saturated LSV curves of Rh SA/GDY under ambient and 55-atm conditions, respectively. (*D*) The NH_3_ yields and their corresponding FEs of Rh SA/GDY under ambient and 55-atm conditions, respectively. (*E*) Comparison of the NH_3_ yield, FE, and *j*_NH3_ for Rh SA/GDY versus the exerted N_2_ pressures. (*F*) Cycling stability results of Rh SA/GDY at −0.2 V. (*G* and *H*) ^1^H NMR spectra and corresponding ^15^NH_4_^+^ yield with the isotopic ^15^N_2_ for 6- and 12-h electroreduction. (*I*) Comparison of the optimum NH_3_ yield, FE, and *j*_NH3_ for the prepared Rh, Ru, and Co SA/GDY.

Nitrogen has poor solubility in water and its concentration in water at standard conditions (25 °C and 1 atm) is 6.1 × 10^−4^ M. Such low N_2_ concentration, accompanied by its sluggish kinetics, induces the low N_2_ adsorption and activation. According to Henry’s law, the dissolved N_2_ concentration in water is in proportion to the N_2_ partial pressure (Fig. 3*A*). It is logical to anticipate that more N_2_ could be delivered to the electrode surface under high N_2_ partial pressure. Thereby, the performance metrics of ENRR were examined in a membrane-separated three-electrode cell under ambient and pressurized conditions, respectively. Under both conditions, a high-purity N_2_ gas (99.999%) was purged sequentially through a Cu-based impurity trap and a liquid-nitrogen-cooled trap to capture the impurities such as NO_*x*_ and residual NH_3_. Under ambient conditions the adequately cleaned ^14^N_2_/^15^N_2_ gas was continuously circulated in the electrolyzer while under pressurized conditions the purified ^14^N_2_ gas was delivered to the electrolyzer without gas circulation (Fig. 3*B* and *SI Appendix*, Figs. S5 and S6). The setups manipulated at different environments hold great promise not only to eliminate the exogenous contaminations but also to reduce the consumption of supplied gas, especially, the expensive ^15^N_2_ gas, thus leading to an accurate and cost-effective electrolysis scheme (2). It should be noted that the anodic compartment in the pressurized electrolyzer contains not only a mixed solution of 0.005 M H_2_SO_4_ and 0.1 M K_2_SO_4_ but also additional 0.01 M ascorbic acid to prevent the oxygen evolution reaction. All of the potentials were recorded versus a Ag/AgCl electrode and converted to the reversed hydrogen electrode (RHE) scale. For a quantitative assessment of electrochemical NH_3_ production, ion chromatography and NMR were explored while the potential by-product of hydrazine was evaluated by the Watt and Chrisp approach. Their corresponding calibration curves are depicted in *SI Appendix*, Figs. S7–S9.

Before starting the pressurized ENRR measurements, we used an internal reference, K_3_[Fe(CN)_6_], to test if the reference electrode could properly work under pressurized conditions. The cyclic voltammetry of the ferri/ferrocyanide redox couple was recorded on a glassy carbon electrode. As shown in *SI Appendix*, Fig. S10, the corresponding redox peaks situated at almost the position despite the exerted pressure changes, ensuring the reliable potentials collected in the following pressurized ENRR measurements. Taking Rh SA/GDY as a representative example, we start with systematic ENRR tests under various N_2_ pressures (1−55 atm N_2_). The linear sweep voltammetry (LSV) curves show an increased response for current density under the elevated N_2_ pressures of 1, 10, 30, and 55 atm, respectively (*SI Appendix*, Fig. S11). Moreover, under the pressure of 55 atm, the LSV curves recorded in the electrolyte with saturated N_2_ and Ar exhibit a more obvious enlarged current gap than those under ambient conditions, presumably contributed by the enrichment of accessible N_2_-induced preferential ENRR (Fig. 3*C*). Next, we conducted pressure-dependent chronoamperometry measurements for Rh SA/GDY at controlled potentials to examine the electroproduction of NH_3_ yield and FE. To our delight, the current densities at different potentials exhibited little degeneration in all electrocatalytic environments, suggesting the robust structure of Rh SA/GDY catalyst (*SI Appendix*, Figs. S12*–*S15*B*). The posttested electrolyte was subjected to ion chromatography, in which the retention time located around 4 min was contributed by NH_4_^+^ (*SI Appendix*, Figs. S12*–*S15*A*). We found that the Rh SA/GDY exhibited a moderate ENRR activity under 1 atm, and the corresponding NH_3_ yield rate and FE gradually increased as the potential is negatively shifted and reached at peak values of 10.10 μg h^−1^⋅cm^−2^ and 4.12% at −0.25 and −0.2 V, respectively (*SI Appendix*, Fig. S12*C* and Table S2). After the thresholds, the relative performance decreased due to the diffusion limitation of N_2_ to the surface of the electrode provoked by vigorous HER. Increasing N_2_ pressure to 10, 30, and 55 atm in the electrolyzer leads to substantially enhanced NH_4_^+^ production, and their optimized performances were achieved as follows: 16.31 μg h^−1^⋅cm^−2^ at −0.25 V and 5.56% at −0.2 V under 10 atm; 36.87 μg h^−1^⋅cm^−2^ and 12.31% at −0.2 V under 30 atm; 74.15 μg h^−1^⋅cm^−2^ and 20.36% at −0.2 V under 55 atm (Fig. 3*D* and *SI Appendix*, Figs. S13–S15*C* and Tables S3–S5). Fig. 3*E* summarizes and compares the optimum NH_3_ yield, FE, and NH_3_ partial current ($j_{NH_{3}}$) under different N_2_ pressures. Clearly, all of the values of NH_3_ yield rate, FE, and $j_{NH_{3}}$ of Rh SA/GDY increased markedly along with the increase of N_2_ pressure. Especially, the NH_3_ yield rate, FE, and $j_{NH_{3}}$under 55 atm render 7.3-, 4.9-, and 9.2-fold enhancements, respectively, than those obtained under ambient conditions. These observations highlight the ENRR activity of Rh SA/GDY under pressurized environments by engaging both N_2_ delivery and HER suppression. As expected, the pressurized ENRR behavior of Rh SA/GDY by far surpasses many of the previously reported state-of-art ENRR catalysts (*SI Appendix*, Table S10). Of note, no hydrazine was detected under the pressurized electrosystem, suggesting the excellent selectivity of the catalyst (*SI Appendix*, Fig. S16).

To verify the nitrogen source of the produced NH_3_ by Rh SA/GDY, we carried out four control experiments and measured the NH_3_ production using 1) the electrolyte pressurized with Ar at −0.2 V for 2 h, 2) the electrolyte pressurized with N_2_ at open-circuit potential for 2 h, 3) the electrolyte pressurized with N_2_ at −0.2 V with bare carbon cloth for 2 h, and (4) the electrolyte saturated with ^15^N_2_ at open-circuit potential for 2 h under ambient conditions. None or a negligible amount of NH_4_^+^ signals were detected in those tests, ruling out the possibility of catalyst degradation or extraneous pollution-induced NH_3_ production (*SI Appendix*, Fig. S17).

Under 55 atm N_2_, we have measured the ENRR stability of the Rh SA/GDY electrodes by carrying out six cycles of electrolysis at −0.2 V. The results showed remarkable stability with negligible attenuation in terms of NH_3_ yield rate and FE. For instance, the NH_3_ production activity remained 88.3% of the initial performance after six runs (Fig. 3*F* and *SI Appendix*, Fig. S18 and Table S6). The durable electrocatalytic behavior reflects the robust composition/structure of Rh SA/GDY. The morphology of densely populated single Rh atoms on GDY is well conserved without aggregation after the stability measurements (*SI Appendix*, Fig. S19 *A* and *B*). Additional XPS study also verified that the structure and the chemical state of Rh were maintained after electrocatalysis (*SI Appendix*, Fig. S19 *C* and *D*).

Isotope labeling experiments using adequately cleaned ^15^N_2_ as feeding gas were conducted to trace the ammonia synthesis under ambient conditions due to the limited pressure of ^15^N_2_ gas. As illustrated in Fig. 3*G*, the ^1^H NMR spectrum of the electrolyte after electrolysis using ^15^N_2_ as the feeding gas displayed a doublet coupling at 7.15 and 7.02 ppm, confirming the generation of ^15^NH_4_^+^. Meanwhile, the typical triplet peaks of ^14^NH_4_^+^, potentially from impurities of electrolyte and catalyst, were not observed. Moreover, the integration area of ^15^NH_4_^+^ signals growth gradually with the increase of electrolysis time. The amounts of produced ^15^NH_4_^+^ after 6- and 12-h electrolysis were quantified to be 56.4 and 108.6 μg, corresponding to the ^15^NH_3_ yield rates of 9.4 and 9.05 μg h^−1^⋅cm^−2^, respectively (Fig. 3*H*); their FEs were calculated as 3.35 and 2.98% (*SI Appendix*, Fig. S20 and Table S7), respectively. These results are very close to the above-mentioned results under 1 atmosphere of ^14^N_2_, implying that the detected NH_3_ in the electrolysis experiments are indeed from the NRR.

Next, it is interesting to see if the pressurized electrolysis can be applied to regulate ENRR performances of other SACs in general. We then proceed with the pressurized ENRR experiments using Ru SA/GDY and Co SA/GDY at 55 atm. As expected, the catalysts displayed fairly high ENRR activity, among which Ru SA/GDY could drive a maximum NH_3_ yield rate of 47.3 μg h^−1^⋅cm^−2^, an FE of 11.76%, and a $j_{NH_{3}}$ of 0.22 mA cm^−2^ at −0.2 V, and the Co SA/GDY gave optimal values of 33.46 μg h^−1^⋅cm^−2^ at −0.3 V, an FE of 8.2%, and a $j_{NH_{3}}$ of 0.13 mA cm^−2^ at −0.25 V (Fig. 3*I* and *SI Appendix*, Figs. S21 and S22 and Tables S8 and S9). The high performances show advantages of the SAC-based NRR and the pressurized electrolysis systems, demonstrating a general and cooperative strategy in reprogramming ENRR. Taken all together, the observations presented in this work showcase the conclusion that nanostructuring of high-loading metal-based SACs in combination with the pressurized electrosystems could suppress the HER, enhance the N_2_ adsorption, and thus improve the ammonia production efficiency of the ENRR in general.

### DFT Calculations.

We next performed first-principles calculations with density-functional theory (DFT) implanted in VASP at the spin-polarized generalized gradient approximation level for Rh SA/GDY to unravel the effects of the pressurized system in boosting ENRR activity (42, 43). The binding energy of Rh atom to GDY is calculated to be −5.37 eV, which suggests that the Rh/GDY catalyst is very stable. Bader analysis shows that 0.38 electron is transferred from Rh to GDY. The projected density of states of Rh and the C atoms coordinated to Rh shows that their orbitals are greatly hybridized, consistent with the strong binding of Rh (*SI Appendix*, Fig. S23). The partial pressure of N_2_ is 50 atm unless otherwise specified. There are potentially two adsorption fashions for N_2_ on Rh SA/GDY: the end-on one and the side-on one. At 50 atm of N_2_, the adsorption free energies of N_2_ are calculated to be −0.48 eV for the former and −0.06 eV for the latter. The end-on N_2_* is more stable than the side-on N_2_*. The adsorption free energy of N_2_ in the end-on fashion at 1 atm is calculated to be −0.38 eV. In comparison, the driving force for the formation of end-on N_2_* at 50 atm is 0.1 eV larger than that at 1 atm, corresponding to 9.62 kJ/mol energy. As a result, the high pressure of N_2_ significantly promotes the adsorption of N_2_, which in turn suppresses the HER. We have also investigated the activity of the alkyne C atoms coordinated to Rh. The adsorption of N_2_ at these C sites is uphill by 2.29 eV at 50 atm, which is 1.39 eV lower than that on the pristine graphdiyne, but 2.77 eV higher than that at the Rh site. Therefore, the Rh site is much more active for the adsorption and reduction of N_2_.

**Fig. 4.**
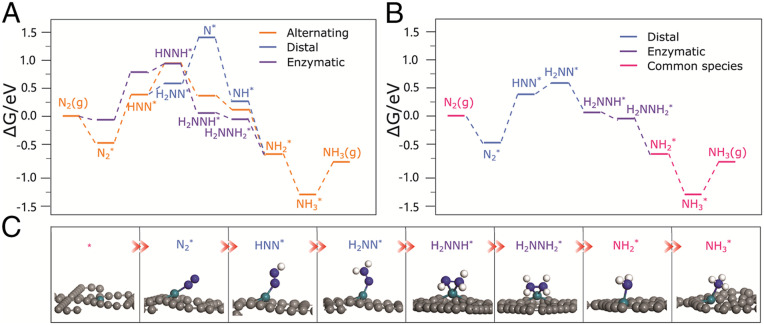
(*A*) Free-energy surfaces for N_2_ reduction following the distal, alternating, and enzymatic pathways. (*B*) The hybrid reaction pathway via the low-energy intermediates. (*C*) The optimized structures of intermediates in Fig. 4*B*. Gray, green, blue, white spheres present the C, Rh, N, H atoms, respectively. Note that in the alternating pathways and the enzymatic pathways, two N are hydrogenated alternatively; the species take side-on adsorption patterns in the alternating and distal pathways but end-on patterns in the enzymatic pathway.

Three possible pathways for the N_2_ reduction were investigated, namely alternating, distal, and enzymatic pathways (44, 45). The calculated free-energy surfaces and the optimized structures are shown in Fig. 4. For both alternating and distal pathways, N_2_ adsorption is in the end-on fashion with the adsorption free energy of −0.48 eV. At 0.0 V vs. RHE, the first hydrogenation step (N_2_*→HNN*) is uphill by 0.86 eV. Following the alternating pathway, HNN*→HNNH* is still endothermic by 0.59 eV. Other hydrogenation steps in this path are all downhill (note that the desorption of NH_3_ is endothermic by 0.58 eV, which is surmountable at room temperature). Therefore, the potential limiting step of the alternating pathway is the first step with a limiting potential of −0.86 V.

The distal pathway shares many common intermediates with the alternating pathway, which includes the end-on adsorbed N_2_* and HNN*, NH_2_* and NH_3_*. Following the distal pathway, the end-on HNN* is not converted to HNNH* but to H_2_NN*. This step is only endothermic by 0.20 eV. With the transfer of the third (H^+^+e^−^), H_2_NN* is reduced to N* with a NH_3_ molecule released. This step is uphill by 0.82 eV, which is slightly lower than that for N_2_*→HNN*. Hydrogenation of N* to NH_3_* is all exothermic. Therefore, the potential-limiting step for the distal pathway is still the first proton-coupled electron step with a limiting potential of −0.86 V.

For the enzymatic pathway, the N_2_ adsorption mode is considered as the side-on fashion with the adsorption free energy of −0.06 eV. Similar to the alternating pathway, two N atoms are also hydrogenated alternatively except that the intermediates take the side-on adsorption patterns. Interestingly, N_2_* and HNN* prefer the end-on adsorption, whereas H_2_NNH* and H_2_NNH_2_* prefer the side-on adsorption (Fig. 4*B*). The energy of the side-on HNNH* is the same as that of end-on HNNH* but 0.39 eV higher than that of H_2_NN* in the distal path. Although the energy of the side-on N_2_* and HNN* are higher than those of the end-on N_2_* and HNN*, the reduction of the side-on N_2_* to HNN* is uphill by 0.85 eV, which is almost the same as that of the alternating pathway. The reduction of the side-on HNN* to HNNH* is slightly endothermic by 0.19 eV. Other hydrogenation steps in the enzymatic pathway are all exothermic. Therefore, the potential-limiting step of the enzymatic pathway is also the first hydrogenation step with a limiting potential of −0.85 V.

Taken all these intermediates into consideration, the N_2_ reduction by Rh SA/GDY would very likely follow a hybrid path via low-energy intermediates (Fig. 4 *B* and *C*) (44, 46). For example, the H_2_NN* species in the distal pathway may shuttle to the enzymatic pathway, which avoids the high-energy intermediate N*, leads to the formation of side-on H_2_NNH*, and thereby reduces the barrier for the conversion of H_2_NN*. But, the potential-limiting step of this hybrid pathway is still N_2_*→HNN* with a limiting potential of −0.86 V. However, if N_2_* in the enzymatic pathway shuttles to the distal pathway first and then H_2_NN* shuttles back to the enzymatic pathway, the limiting potential will become −0.44 V.

## Conclusion

In summary, we have demonstrated positive cooperation of metal single-atom catalysts with a pressurized electrolysis system to simultaneously regulate both kinetics and thermodynamic process, leading to a significant boost in the ENRR activity by delivering more N_2_ to the electrode surface and thus impeding the HER. The applied catalysts were nanostructured by stereoconfinement-induced single-metal atoms (Rh, Ru and Co) on graphdiyne, and these catalysts feature ultrahigh loading amounts of isolated metal centers. The pressurized ENRR measurements showcased significantly enhanced ammonia electroproduction with simultaneously reduced HER. Under pressurized conditions of 55 atm of N_2_, the Rh SA/GDY displayed a NH_3_ yield rate of 74.15 μg h^−1^⋅cm^−2^, an FE of 20.36%, and a *j*_NH3_ of 0.35 mA at −0.2 V, which values are 7.3-, 4.9-, and 9.2-fold higher than those obtained at the ambient conditions. The proposed strategy and findings provide a valuable electrocatalysis scheme that cooperatively integrates both efficient catalysts and the pressurized reaction system, which is a step forward to the electrochemical ammonia production.

## Materials and Methods

The synthetic strategy and experimental sections of this work are provided in *SI Appendix*. These methods contain the synthesis of Rh SA/GDY, Ru SA/GDY, and Co SA/GDY catalysts, structure characterization. Detailed electrochemical measurements under ambient and pressurized conditions are also included. The calculation method for NH_3_ yield rate and FE and the computation theory are elaborated in *SI Appendix*.

## Supplementary Material

Supplementary File

## Data Availability

All study data are included in the article and *SI Appendix*.
