# Supplementary material for: Regulating kinetics and thermodynamics of electrochemical nitrogen reduction with metal single-atom catalysts in a pressurized electrolyser
Source: Proc Natl Acad Sci U S A. 2020 Nov 10;117(47):29462–8. doi: 10.1073/pnas.2015108117 (PMC7703585; doi:10.1073/pnas.2015108117)
Supplement: Supplementary File [file pnas.2015108117.sapp.pdf]

PNAS
www.pnas.org

Supplementary Information for

Regulating kinetics and thermodynamics of electrochemical nitrogen reduction with metal single-atom catalysts in a pressurized electrocatalytic electrolyser

Haiyuan Zou[a,b], Weifeng Rong[a], Shuting Wei[a], Yongfei Ji[c]* and Lele Duan[a,d]*

Yongfei Ji, Lele Duan
Email: yongfeiji2018@gzhu.edu.cn；duanll@sustech.edu.cn;

This PDF file includes:

Experimental sections
Figs. S1 to S23
Tables S1 to S10
SI References

www.pnas.org/cgi/doi/10.1073/pnas.2015108117

Experimental Section

Synthesis of Hexakis[(trimethylsilyl)ethynyl]benzene（HEB）. The pristine HEB was prepared according to the previous method developed by the Li group in 2010.[1] To a Schlenk flask containing trimethylsilylacetylene (40 mmol) and dry tetrahydrofuran (30 mL) at –78 ^{o}C was added *n*-butyllithium (1.6 M, 40 mmol) and stirred for 60 min. Then, a solution of zinc chloride in THF (0.5 M, 80 mL) was added dropwise to the reaction mixture at –78 ^{o}C, and then allowed to reach room temperature and stirred overnight. The reaction mixture was filtered under an argon atmosphere and cannulated to a 250 mL three-necked flask containing a suspension of hexabromobenzene (4 mmol, 2.21 g) and tetrakis(triphenylphosphine)palladium(0) (1 g, 0.8 mmol) in dry tetrahydrofuran (50 mL) at 20 ^{o}C. The reaction mixture was additionally stirred under an argon atmosphere at 80 ^{o}C for 3 days

After the addition of 100 mL of 0.1 M aqueous HCl, the reaction mixture was extracted with ethyl acetate. The combined organic phases were washed with brine and dried over magnesium sulfate. The solvent was evaporated and further purified by chromatography on silica gel (eluent hexane/dichloromethane = 6:1) to give the crude hexakis[(trimethylsilyl)ethynyl]benzene as a brown solid. After recrystallization in ethyl acetate or hexane twice, the product was obtained as pale crystals (1.36 g, 52.1%). ^{1}H NMR (400 MHz, $CDCl_3$, 7.26 ppm, 25 ^{o}C): δ 0.28 ppm (s, $Si(CH_3)_3$). ^{13}C{^{1}H} NMR (100 MHz, $CDCl_3$, 77.16 ppm, 25 ^{o}C): δ 128.10 (s, Ar C), 105.35 (s, Ar-*C*≡C), 101.13 (s, Ar-C≡*C*), 0.17 ppm (s, $Si(CH_3)_3$). ^{29}Si{^{1}H} NMR (79.5 MHz, $CDCl_3$, 25 ^{o}C): δ –17.07 ppm (s, $Si(CH_3)_3$).

Synthesis of M SA/GDY. These catalysts were synthesized under a dry and oxygen-free argon atmosphere by using the Schlenk technique. To a mixture of 43.6 mg (0.066 mmol) hexakis[(trimethylsilyl)ethynyl]benzene and 0.01 mol of rhodium chloride, ruthenium chloride and cobaltous chloride in 15 mL dry pyridine was added dropwise 0.4 ml TBAF (1 M in THF, 0.4 mmol) and stirred at ambient condition for 30 min, and then the solution was heated at 60 ^{o}C for 24 h. Then the subsidence was separated by centrifugation, washed by acetone, ethanol, water and freeze-dried to yield a black powder.

Characterization. Powder X-ray diffraction patterns were collected using a Rigaku diffractometer equipped with Cu Kα radiation (λ =0.15418 nm). Raman spectra were recorded on a Horiba LabRam Aramis HR Evolution confocal Raman spectrometer with a 532 nm laser. X-ray photoelectron spectroscopy was measured on a XSAM800 using Al Kα radiation (1486.6 eV). The binding energies were calibrated to the referencing C 1s to 284.6 eV. Inductively coupled plasma mass spectrometry (ICP-MS) was taken on an Agilent 7700x to determine the metal loading ratio. Transmission electron microscopy, high-angle annular dark-field scanning transmission microscopy and energy-dispersive X-ray spectra were performed on a FEI Titan Themis aberration-corrected transmission electron microscope at 300 kV. Ion chromatography was conducted using a Shine CIC-D100 plus using the dual temperature heater, injection valve, conductivity detector, AERS 500 Anions suppressor. UV-Vis spectra were acquired on an Agilent Technologies Cary 8454. The pH value of the electrolyte was recorded by a pH meter (PHS-3C, Shanghai Yidian Scientific Instrument Co., Ltd). ^{1}H nuclear magnetic resonance (NMR) spectra were collected on a superconducting-magnet NMR spectrometer (Bruker AVAMCE AV III 600). X-ray absorption fine structure (XAFS) measurements were carried out at the beamline 14W1 in Shanghai Synchrotron Radiation Facility (SSRF), China. The storage ring of SSRF was operated at 3.5 GeV with a current 300 mA. Rh foil was used as energy standard, and the as-obtained data was analyzed by Athena and Artemis software.

Electrochemical tests. Electrocatalytic N_2 reduction was assessed under ambient and pressurized conditions independently, and all the electrochemical measurements were performed at room temperature. For both situations, the electrochemical measurements were conducted in a three-electrode H-type cell separated by Nafion 117 membrane. The configuration contains an Ag/AgCl and a platinum mesh electrode, which served as the reference- and counter- electrode, respectively. It should be noted that a small hole was drilled on the reference electrode to keep the balance of relative pressure between the inner and outer. Moreover, to probe the potential pressure effect on the reference electrode, a redox reaction of Ferri/Ferrocyanide on a glassy

carbon (GC) electrode was carried out under the pressurized environment. Typically, under different applied pressures, a cyclic voltammetry measurement was performed in a freshly prepared redox electrolyte containing 0.4 mM $K_3[Fe(CN)_6]$ and 0.5 M KCl aqueous solution using a pre-cleaned GC electrode with a scan rate of 50 mV/s, the corresponding redox potentials were recorded and compared. The working electrode was prepared as follows: 5 mg of the synthesized catalysts were dispersed in 970 μL ethanol and ground with 30 μL Nafion solution (5%, Sigma-Aldrich) for 1 h sonication. Then, 200 μL of the catalyst dispersions were drop-casted onto a pre-cleaned carbon cloth with a geometric area of 1 cm^2 and dried at room temperature. A mixed solution of 0.005 M H_2SO_4 and 0.1 M K_2SO_4 was used as a cathodic electrolyte, while another 0.01 M ascorbic acid was added in such mixed solution for anodic electrolyte, which prevents the oxygen evolution during electrolysis.

The supplied gas was purified before purging into the electrolyzer. In brief, an N_2 stem such the $^{14}N_2$ (99.999%) and $^{15}N_2$ (>99.9%, Cambridge isotope laboratories, Inc.) were passed through a copper impurity trap and a cold trap to capture the NO_x and NH_3 residue. The copper impurity trap was filled with 10 g and 1.5 kg Cu-SAPO catalyst in ambient and pressurized electrocatalysis system, respectively, which was annealed at 300 ^{o}C for 4 h with 5% H_2/Ar gas and additional 1 h with Ar gas, before each electrochemical test. The temperature of the cold trap was −100 ^{o}C, which was cooled by ethanol and liquid nitrogen slurry. All the electrochemical potentials were against to the reversible hydrogen electrode (RHE) scale using: E_{RHE} (V) = $E_{Ag/AgCl}$ + 0.059 pH + 0.197.

For ambient conditions, the purified N_2 gas was flowed into the gas-circulating set-up and continuously resupplied to the electrolyzer by a gas pump. Polarization curves were recorded at N_2- and Ar-recycled electrolyte with the scan rate of 10 mV s^{-1}. Chronoamperometry measurements were conducted at controlled potentials in the N_2-saturated electrolyte for 2 h.

For the pressurized conditions, the aforementioned gas-tight H-cell was transferred into a pressured autoclave (Figure S5). Before measurements, purified N_2 gas was slowly pressured to the system and then degassed, and the procedures were processed for three times to expel the air in the electrolyte and reactor. Thereafter, specific pressures of above-purified N_2 (10 atm, 30 atm and 55 atm) were pressurized to the autoclave and stirred for 1 h to dissolve N_2. Polarization curves were recorded at Ar- and N_2-pressurized electrolytes. The chronoamperometry measurements were tested at different potentials and the consequent electrolyte was directly injected into the ion chromatography to determine the NH_3 yield rate. The durability experiments were evaluated by consecutive cycling tests at 55 atm.

Faraday efficiency and ammonia yield rate. The Faraday efficiency was considered as the proportion of the electric charge for nitrogen reduction to the total charge cross past the circuit. The Faradaic efficiency for the product was obtained using the following formula:

$$FE = N \times C_{NH3} \times F \times V/ (17 \times Q) \times 100\% \quad (1)$$

Where F is Faraday's constant (96485 C mol^{-1}), N is the number of electrons transferred (here N = 3), V is the volume of cathodic electrolyte, C_{NH3} is the measured NH_3 concentration, and Q represents the total electric charge.

The NH_3 yield rate was obtained as follow:

$$v_{NH3} = C_{NH3} \times V/T \times S \quad (2)$$

Where S represents the support area of the catalyst on carbon cloth, T is the electrolysis time.

Ammonia quantification. The concentration of the produced ammonia after each electroreduction tests were scrutinized by the ion chronograph and/or the nuclear magnetic resonance (NMR). Through ion-chronograph, 1.0 mL of the post-electrolyzed electrolytes were filtered through a nylon membrane filter (220 nm) and injected directly to the ion-chronograph. The peak of NH_4^+ was centered at 4 min. The calibration curves were built by a series of standard solutions with appropriate ammonia sulfide concentrations.

For NMR measurements, 450 μL of collected catholyte after pressurizing ENRR test was blended with 50 μL D_2O containing 8 ppm (m/m) dimethyl sulphoxide (≥ 99.9%; Alfa Aesar) as the internal standard. Then, the ^1H spectrum was recorded with water suppression using a Bruker AVIII 600 MHz NMR spectrometer. The calibration curves were established by a series of

standard solutions with appropriate $^{15}NH_4Cl$ (>99.9%, Cambridge isotope laboratories, Inc.) concentrations.

Hydrazine quantification. The concentration of hydrazine in the post-electrolyzed electrolyte was determined by Watt and Chrisp method. Briefly, a color reagent was prepared by blending 5.99 g para-(dimethylamino) benzaldehyde and 30 mL concentrated HCl in 300 mL ethanol. After the chronoamperometry tests, 2.0 mL of the electrolyte was extracted and mixed with 2.0 mL of the color reagent, and then the UV-Vis absorption spectrum was collected after standing for 30 min. The calibration curves were calibrated using standard hydrazine solution assay.

Isotope-labeled experiments

Before the test, the electrocatalytic system was fed with purified Ar for 0.5 h. Then, around 260 mL of purified $^{15}N_2$ gas was purged into the gas circulation set-up, and continuously resupply to the cathodic zone. The electrocatalytic test was recorded at –0.25 V with Rh SA/GDY under ambient conditions, and the concentration of $^{15}NH_4^+$ in the post-tested electrolyte was identified and quantified by ^1H NMR spectroscopy.

Density Functional Theory (DFT) Calculations. Perdew−Burke−Ernzerhof exchange−correlation functional and projector-augmented-wave pseudopotential were adopted.[2, 3] An energy cutoff of 460 eV was applied for the plan-wave basis set. Graphdiyne was described by a monolayer slab with a vacuum layer of 15Å. A 2×2 supercell was used with the Brillouin zone sampled by a 3×3 grid of Monkhorst-Pack k-points.[4] The dispersive interaction was treated with the DFT+D3 scheme.[5] All atoms are allowed to relax until the maxima force on the atoms was smaller than 0.02 eV/Å. Free energy surfaces for N_2 reduction was calculated according to the computational hydrogen electrode, in which the energy of a (H^+ + e^-) was related to the energy of H_2 gas and the applied potential.[6] Free energy corrections (including the zero-point energy, enthalpy, and entropy contributions) were included in the harmonic approximation.[7] The gas-phase molecules were treated as ideal gases.

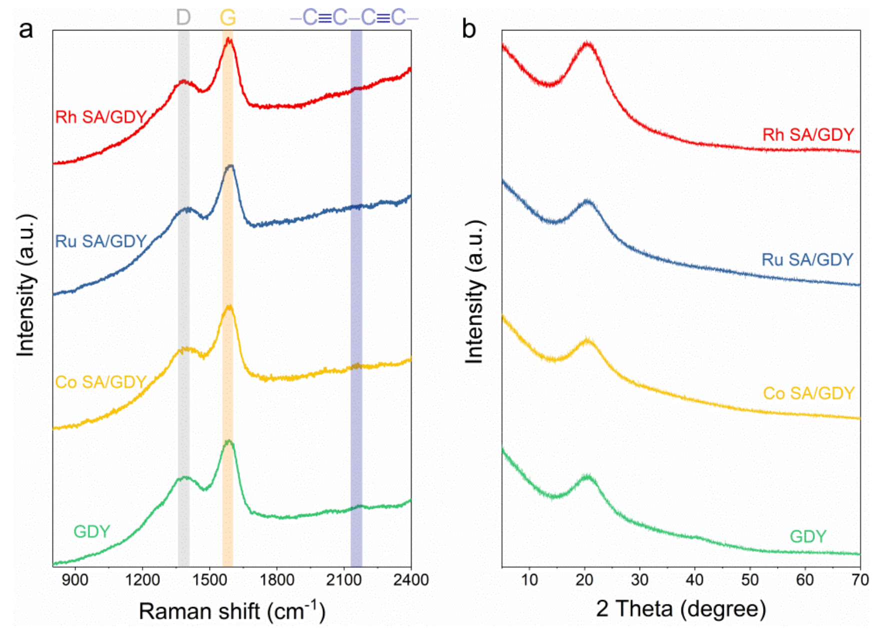

Figure. S1. (a) Raman spectra and XRD patterns of as-prepared Rh SA/GDY, Ru SA/GDY, Co SA/GDY and bare GDY.

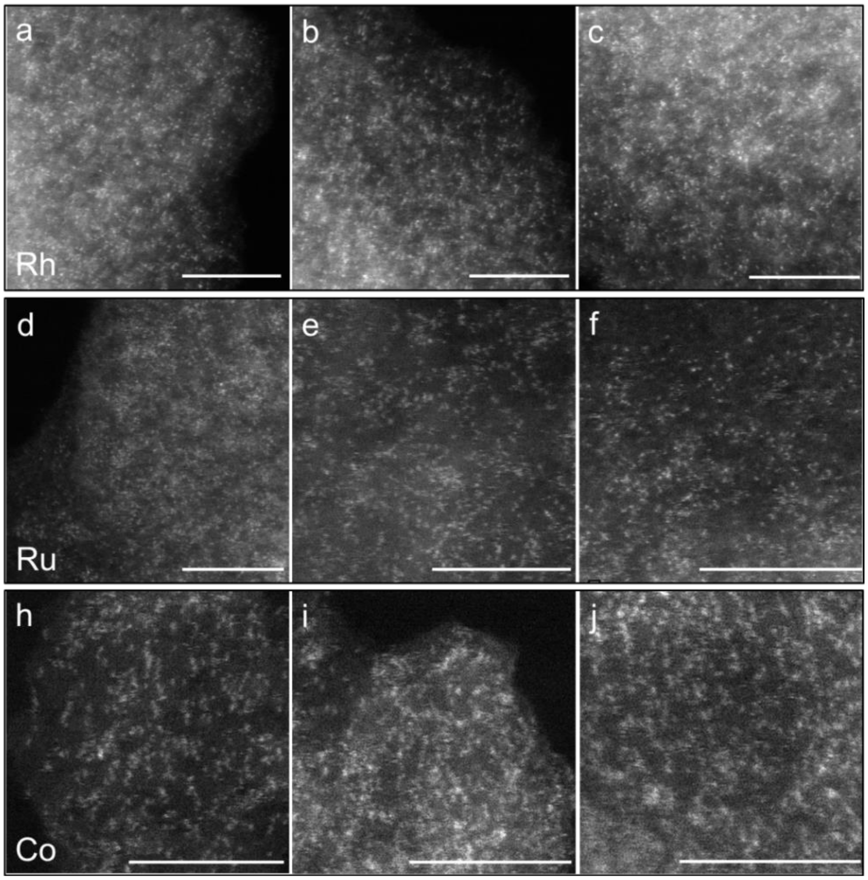

Figure S2. HAADF-STEM images of the prepared samples in different regions. (a-c) Rh SA/GDY. (d-f) Ru SA/GDY. (h-i) Co SA/GDY. Scale bar is 5 nm.

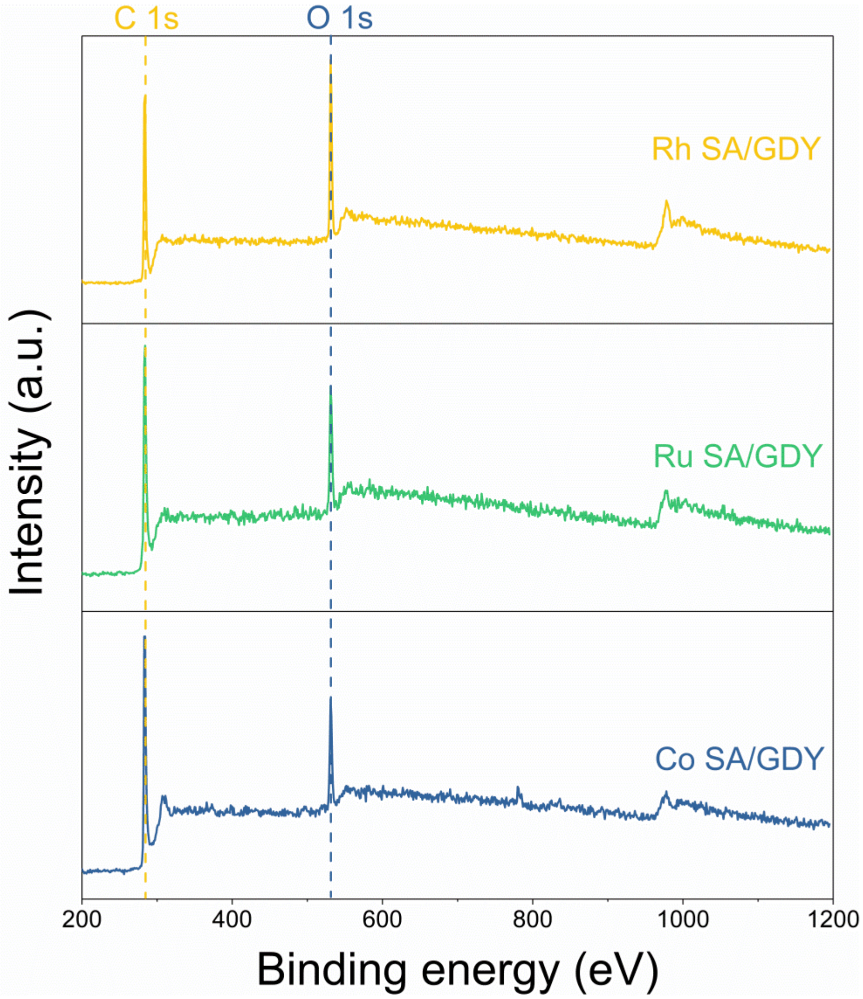

Figure S3. XPS survey spectra of Rh SA/GDY, Ru SA/GDY and Co SA/GDY.

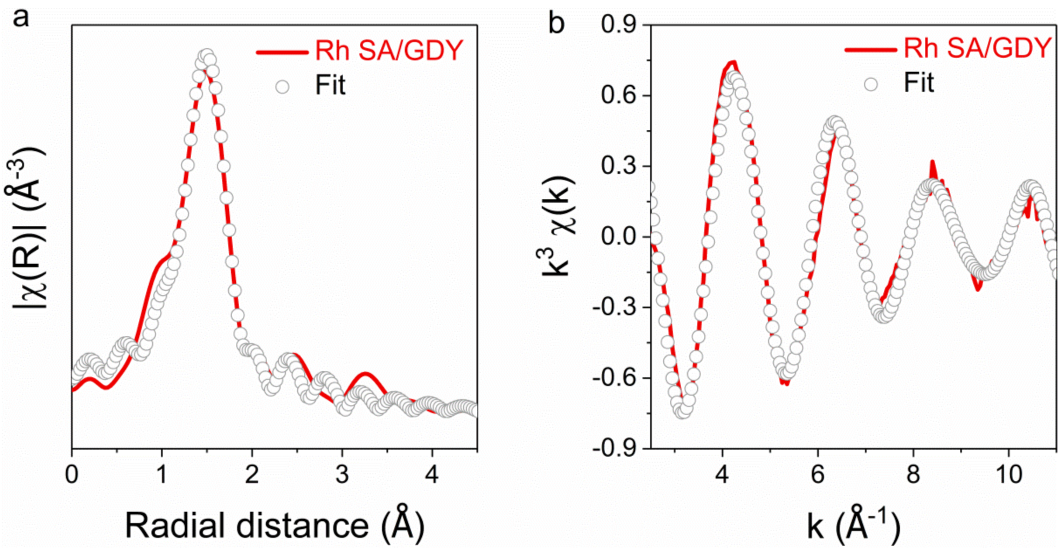

Figure S4. Rh K-edge EXAFS spectra in (a) R space and (b) k space for Rh SA/GDY with the fitted result.

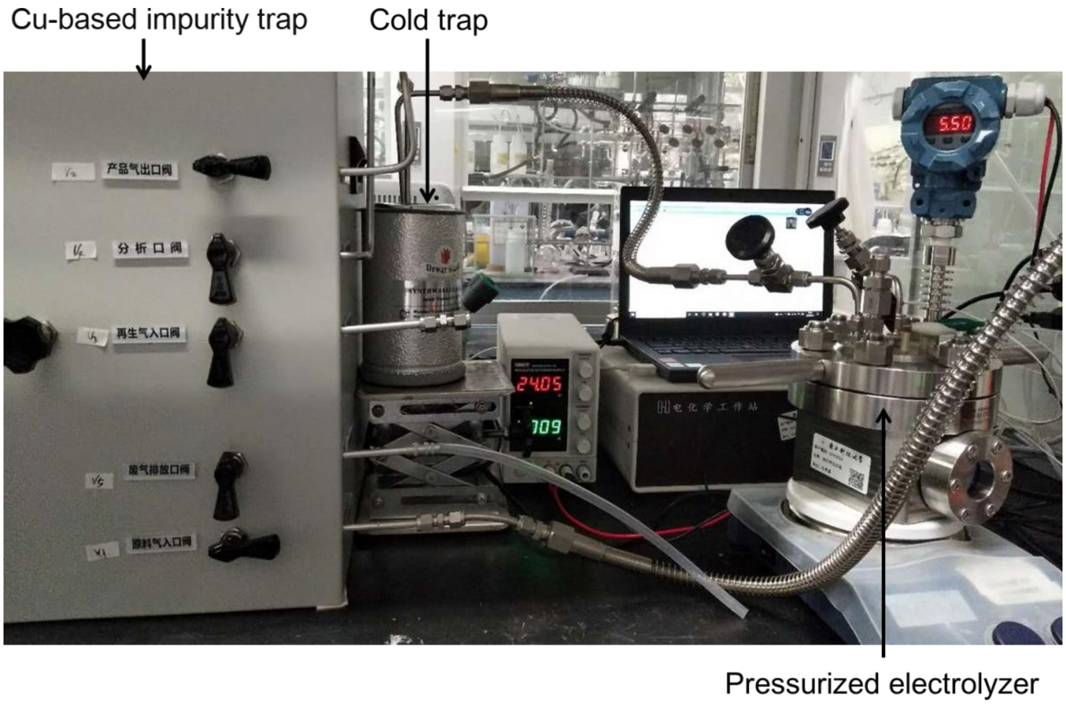

Figure S5. Photograph of the set-up for pressurized N_2 electrolysis.

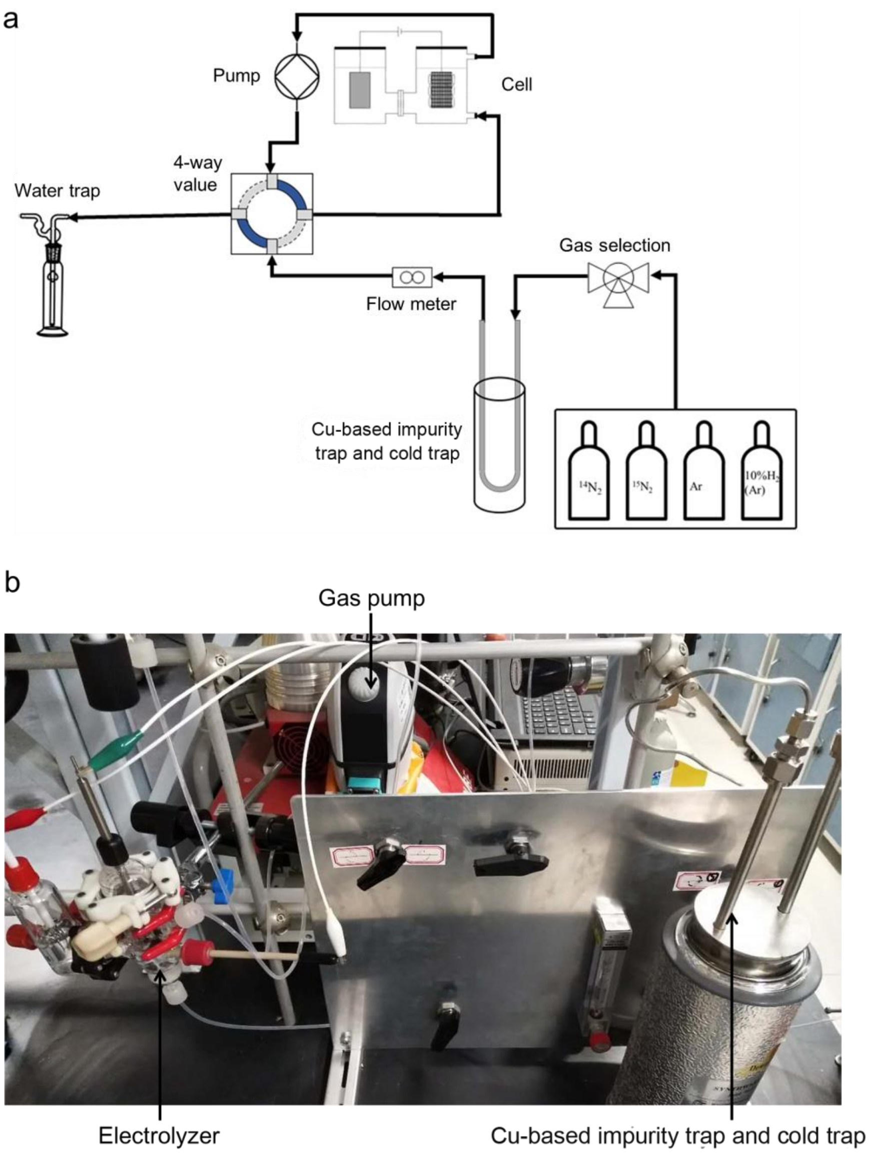

Figure S6. (a) Schematic of the set-up for N_2 electroreduction under ambient conditions. (b) Photograph of the corresponded set-up.

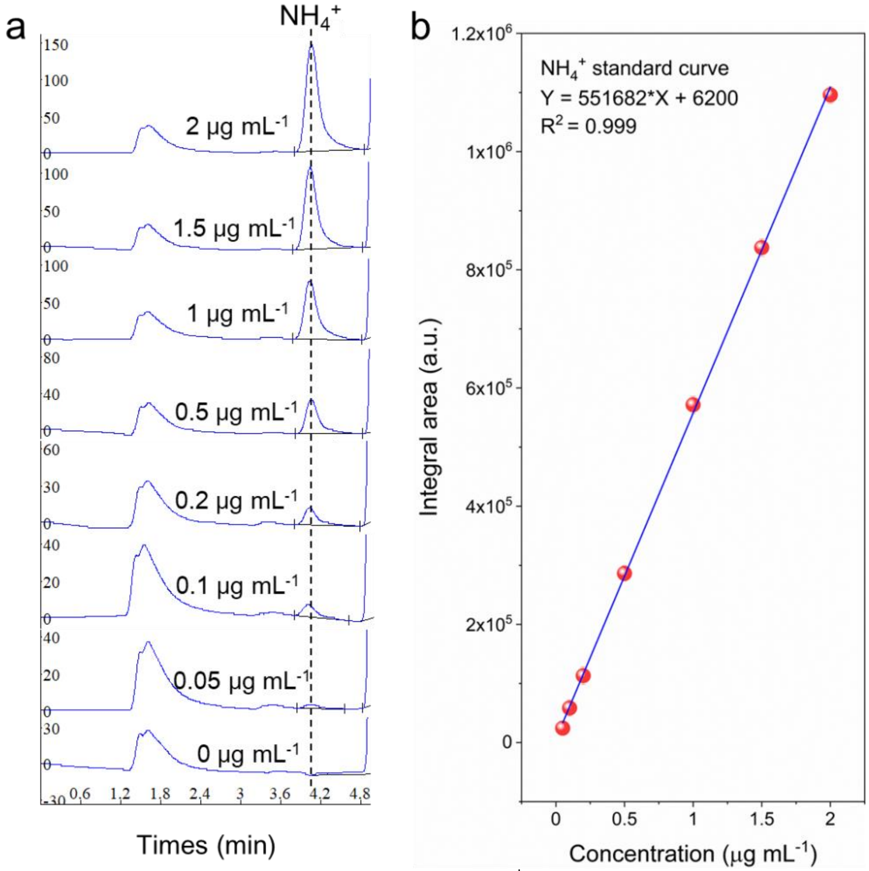

Figure S7. (a) Ion chromatography spectra of NH_4^+ ions with different concentrations. (b) Corresponded calibration curve for NH_4^+.

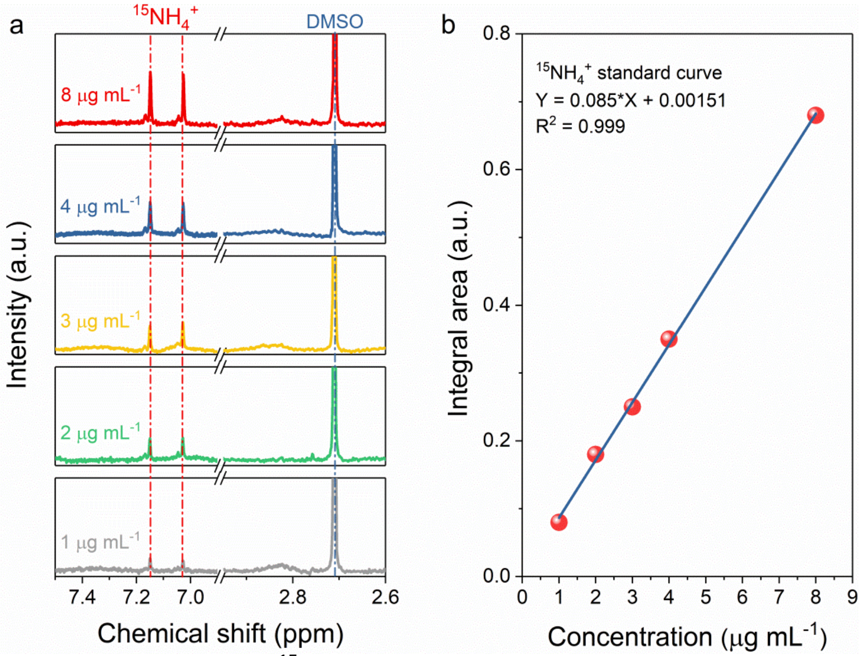

Figure S8. (a) 1H NMR spectra of $^{15}NH_4^+$ with various concentrations, where 8 ppm of dimethyl sulphoxide was used as an internal standard.

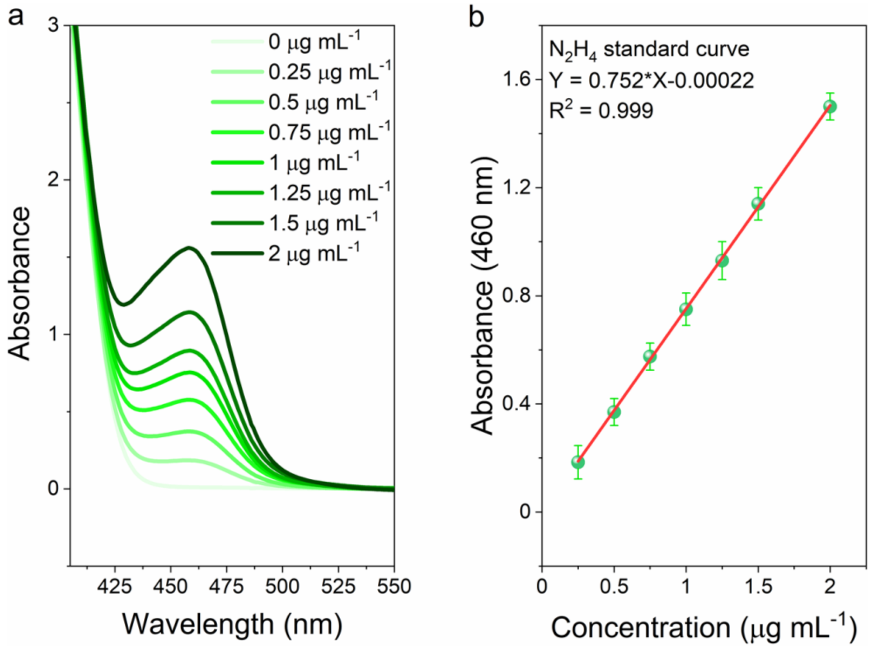

Figure S9. N_2H_4 quantification via Watt and Chrisp method. (a) UV-Vis absorption spectra for various concentrations of N_2H_4. (b) The corresponded calibration curves.

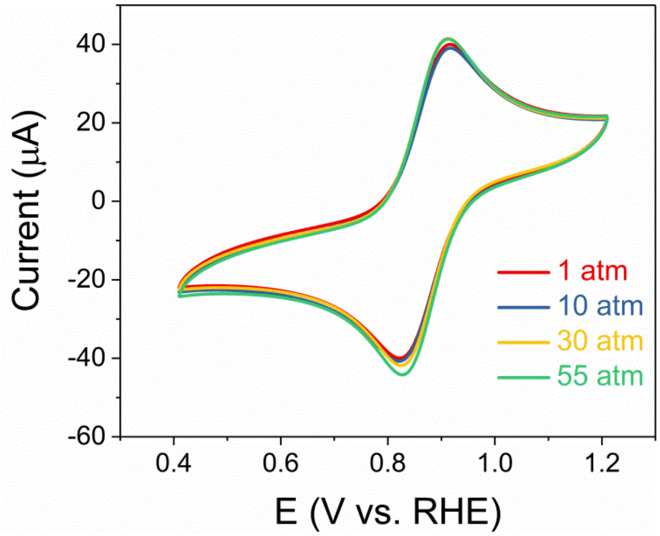

Figure S10. Cyclic voltammetry curves of the glassy carbon electrode in 0.004 M $K_3[Fe(CN)_6]$ and 0.5 M KCl aqueous solution under different applied pressures.

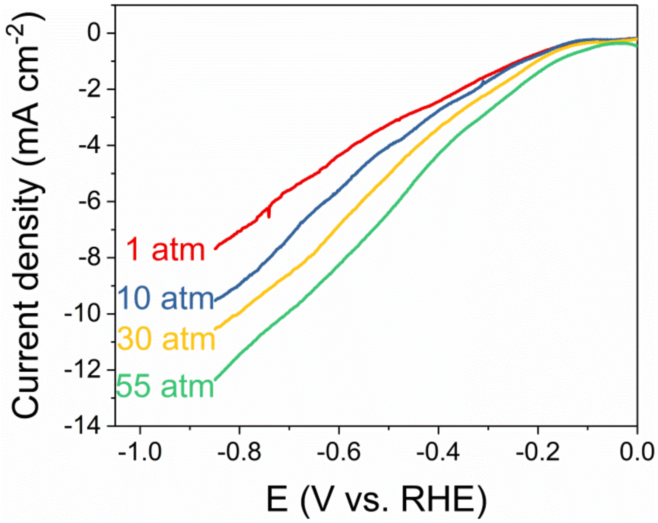

Figure S11. LSV curves of Rh SA/GDY under 1–55 atm N_2 pressures.

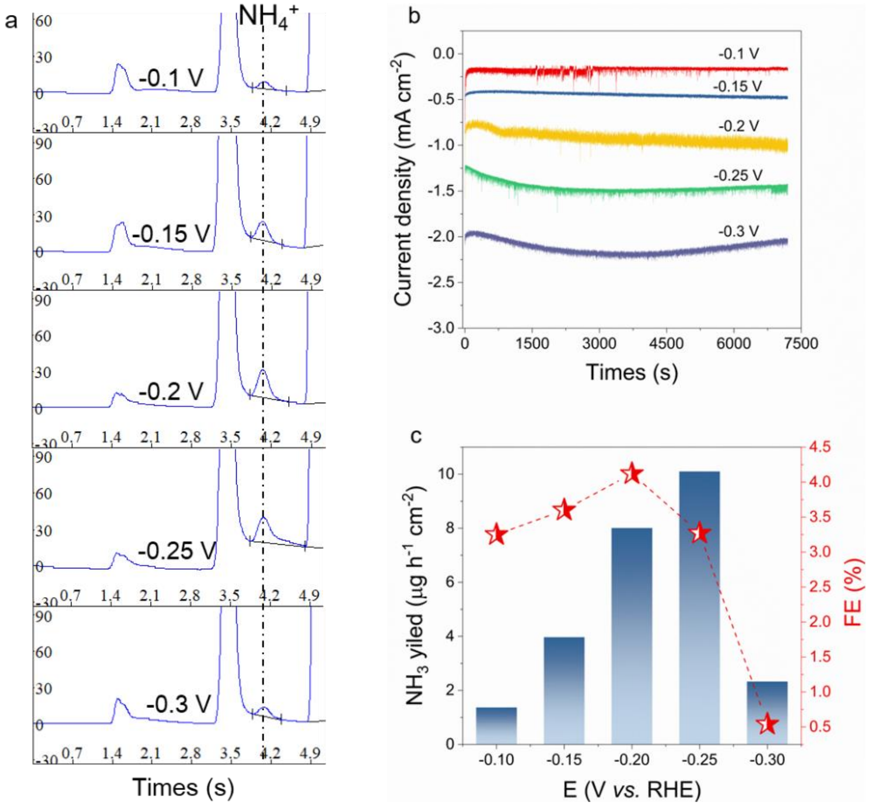

Figure S12. ENRR performance of Rh SA/GDY under ambient conditions. (a) Ion chromatography spectra recorded at different potentials. (b) Chronoamperometric curves at different applied potentials. (c) Corresponded NH_3 yield rate (left axis) and FE (right axis).

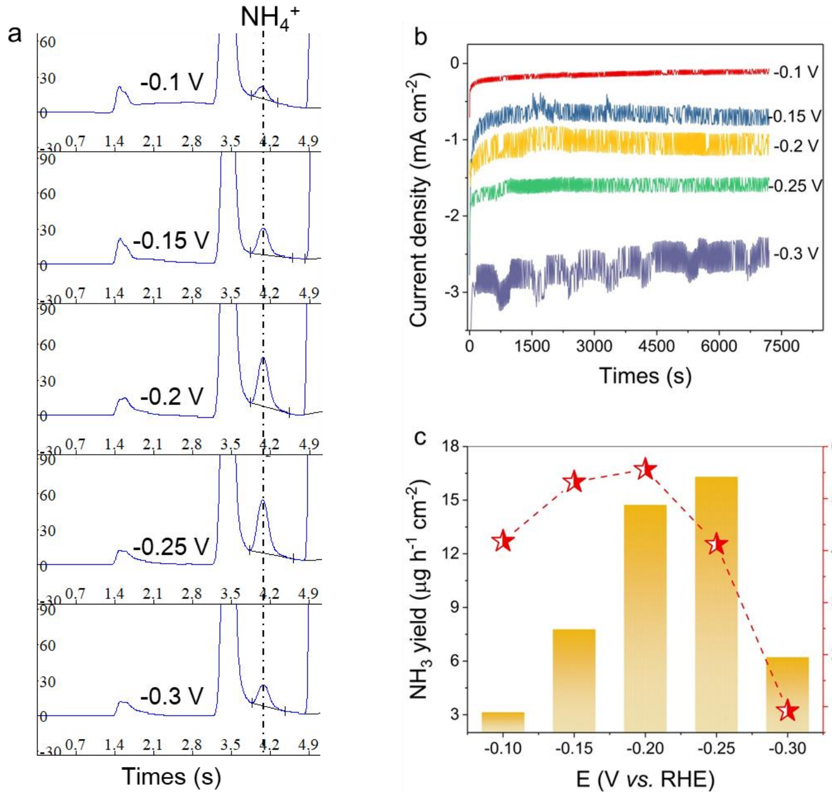

Figure S13. ENRR performance of Rh SA/GDY at N_2 partial pressure of 10 atm. (a) Ion chromatography spectra recorded at different potentials. (b) Chronoamperometric curves at different applied potentials. (c) Corresponded NH_3 yield rate (left axis) and FE (right axis).

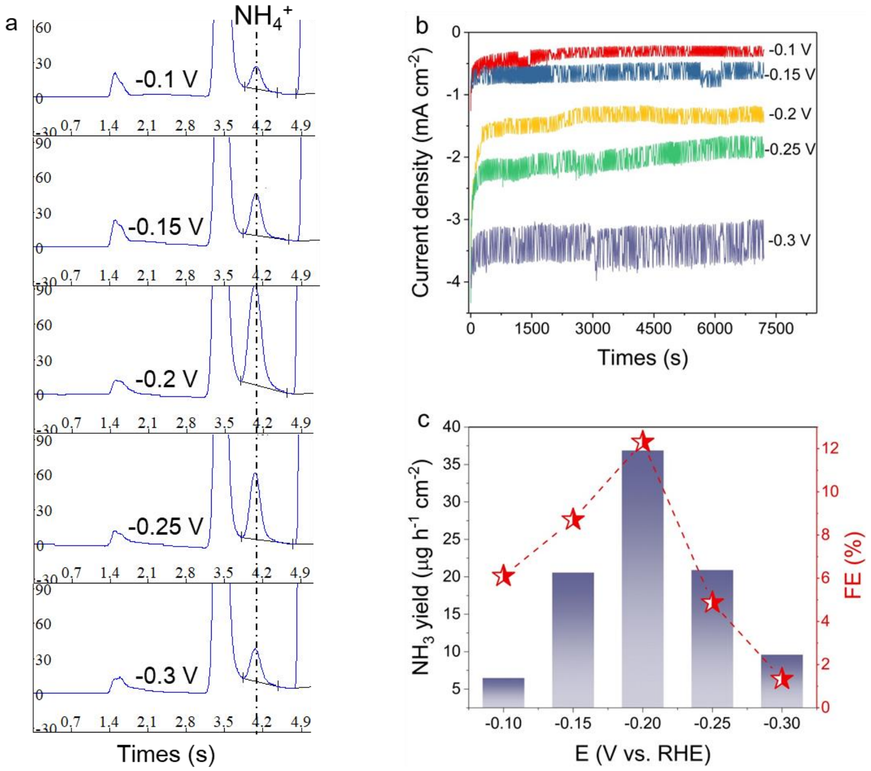

Figure S14. ENRR performance of Rh SA/GDY at N_2 partial pressure of 30 atm. (a) Ion chromatography recorded spectra at different potentials. (b) Chronoamperometric curves at different applied potentials. (c) Corresponded NH_3 yield rate (left axis) and FE (right axis).

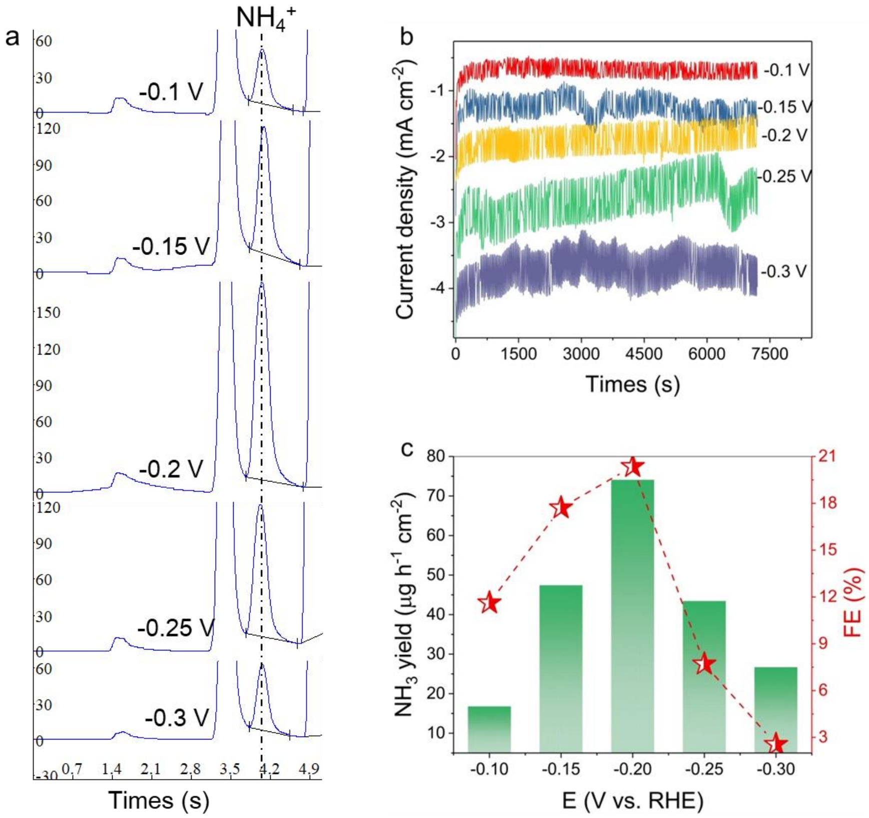

Fig S15. ENRR performance of Rh SA/GDY at N_2 partial pressure of 55 atm. (a) Ion chromatography recorded spectra at different potentials. (b) Chronoamperometric curves at different applied potentials. (c) Corresponded NH_3 yield rate (left axis) and FE (right axis).

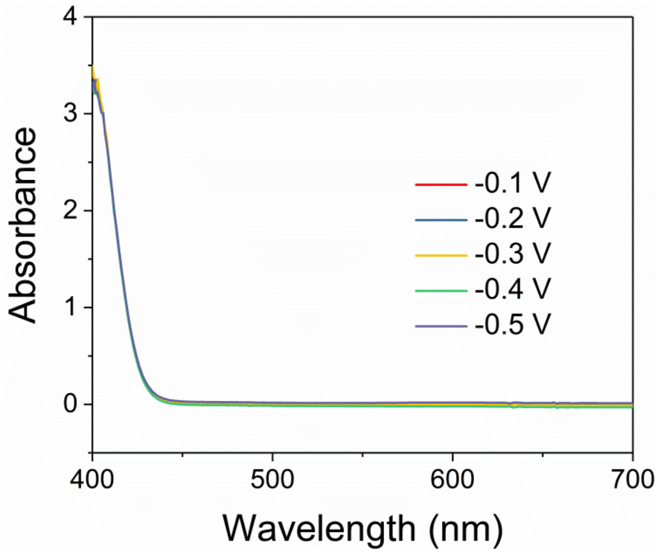

Fig S16. UV-Vis absorption spectra of the electrolytes tested at different potentials under 55 atm N_2 partial pressures.

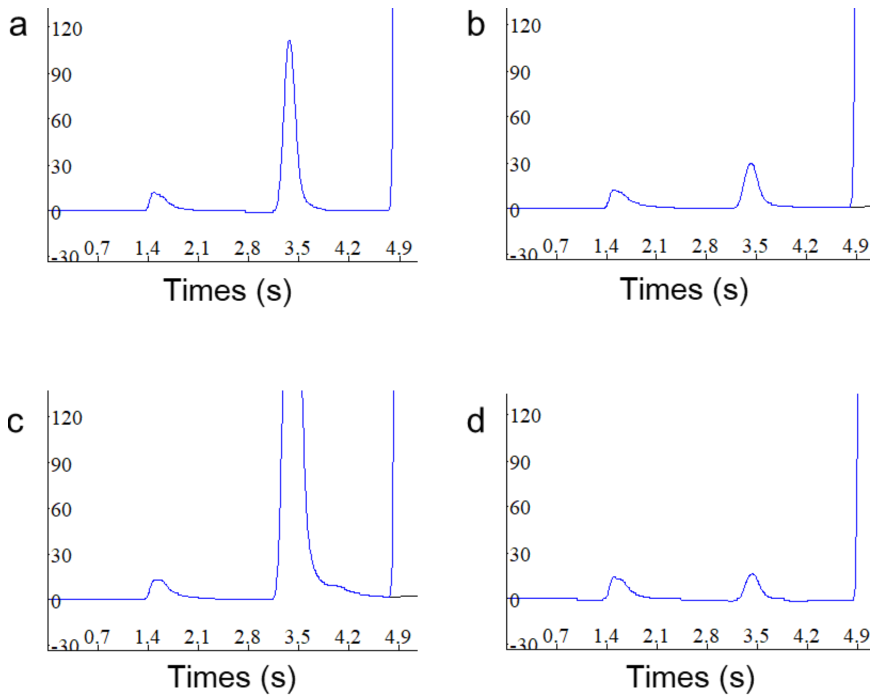

Figure S17. Ion chromatography spectra of four comparative experiments. (a) Ar-saturated electrolyte at an applied potential of –0.2 V under 55 atm for 2 h. (b) N_2-saturated electrolyte at open circuit potential under 55 atm for 2h. (c) N_2-saturated electrolyte at –0.2 V using bare carbon cloth as a working electrode under 55 atm for 2h. (d) $^{15}N_2$-saturated electrolyte at open circuit conditions under ambient conditions for 2 h.

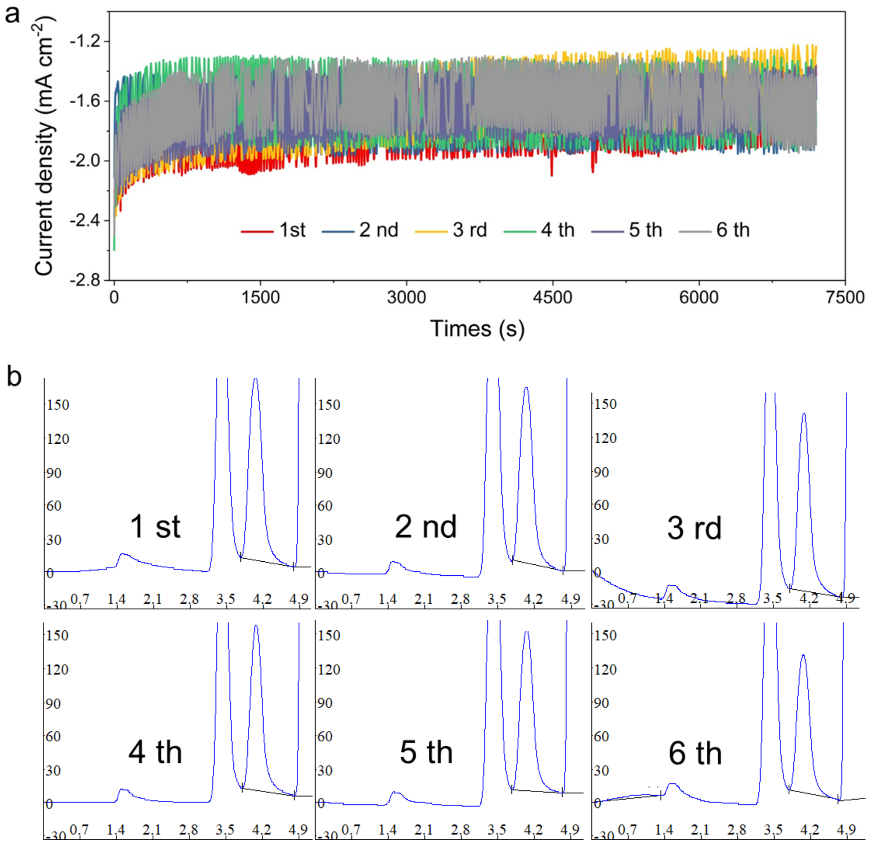

Figure S18. (a) Chronoamperometry results of Rh SA/GDY for cycle stability test recorded at –0.2 V in N_2-pressurized electrolyte at 55 atm. (b) Corresponding ion chromatography spectra of the each cycled electrolyte.

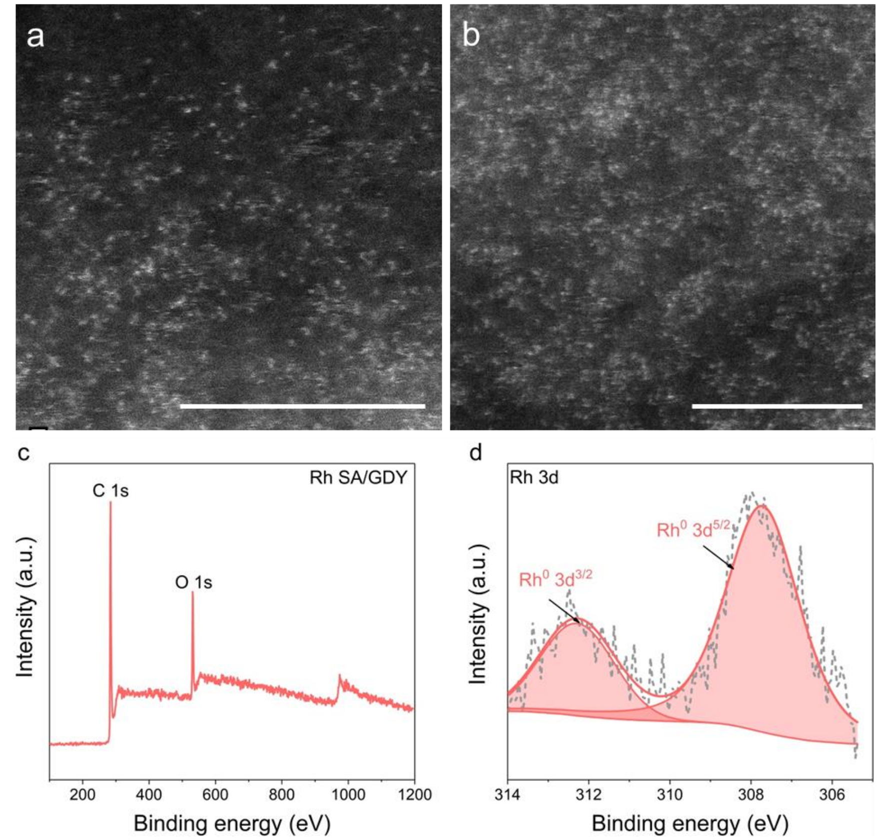

Figure S19. Morphologies and structure analysis of the tested Rh SA/GDY. (a, b) HAADF-STEM characterization of Rh SA/GDY after the ENRR test. Scale bar 5 nm. XPS survey (c) and Rh 3d (d) spectra of the post-tested Rh SA/GDY.

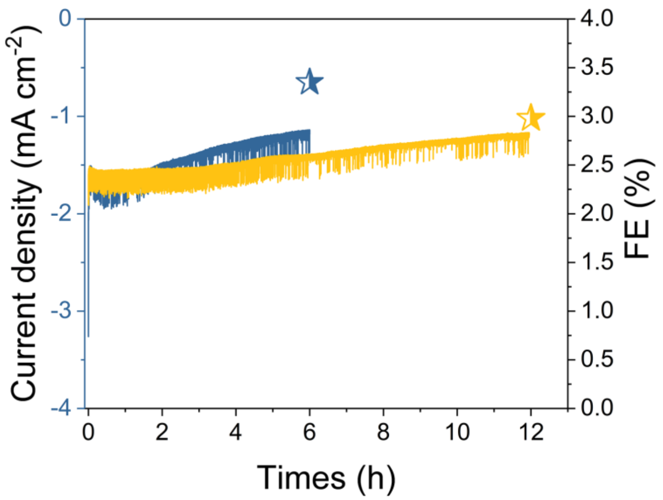

Figure S20. (a) Chronoamperometry results (left axis) of Rh SA/GDY recorded at –0.2 V for different time using $^{15}N_2$ as feeding gas under ambient conditions and the corresponded FE (right axis).

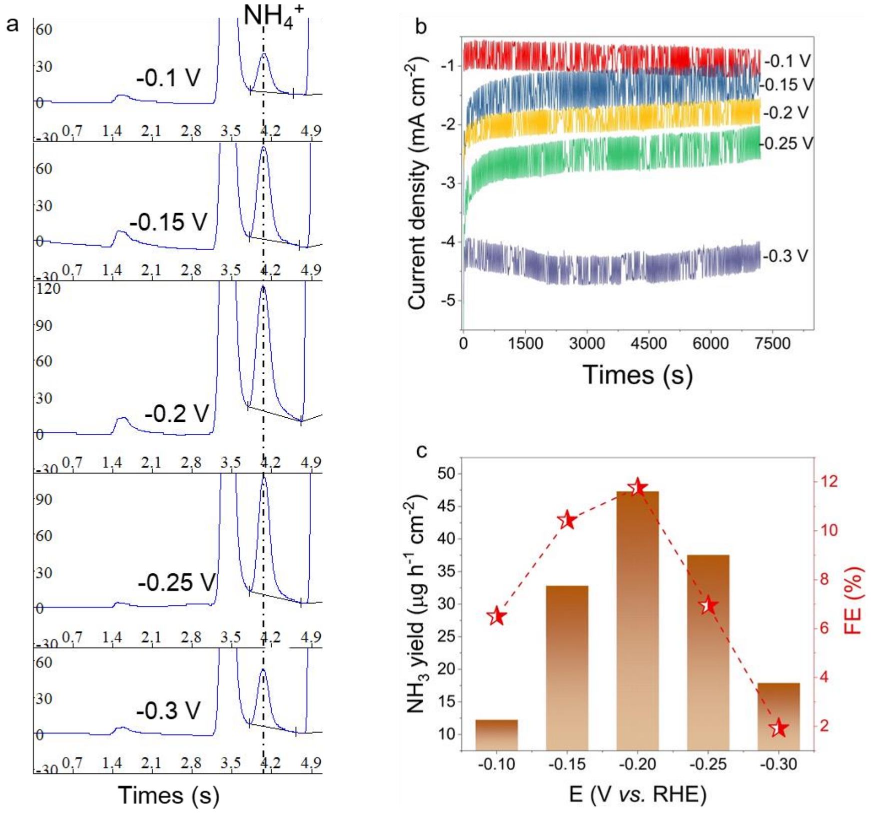

Figure S21. ENRR performance of Ru SA/GDY at N_2 partial pressure of 55 atm. (a) Ion chromatography recorded spectra at different potentials. (b) Chronoamperometric curves at different applied potentials. (c) Corresponded NH_3 yield rate (left axis) and FE (right axis).

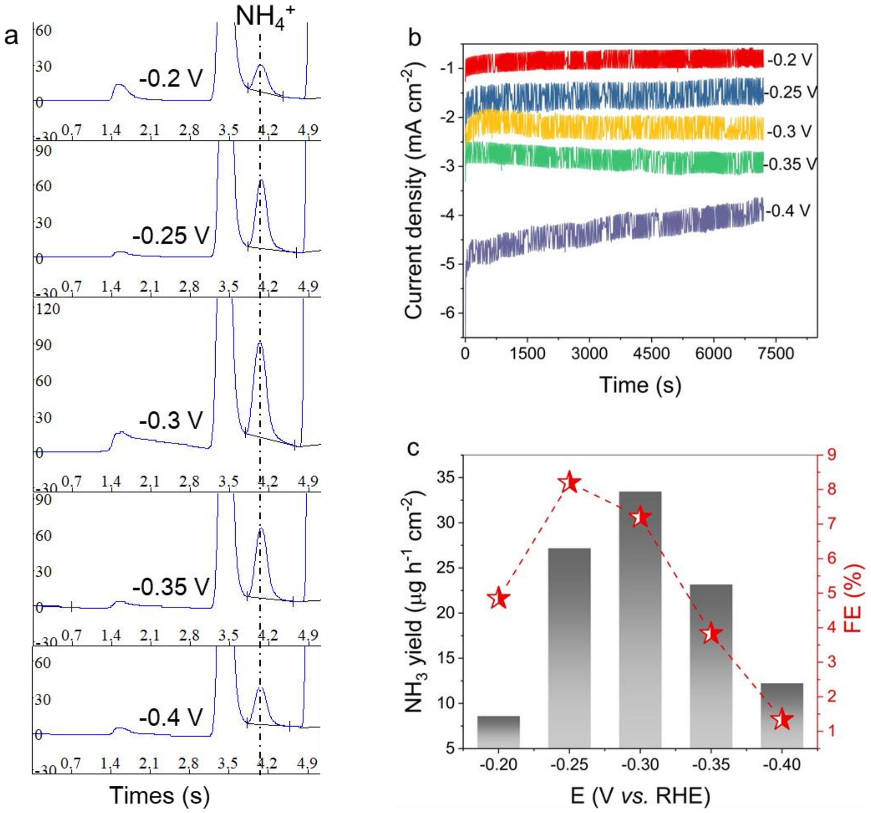

Figure S22. ENRR performance of Co SA/GDY at N_2 partial pressure of 55 atm. (a) Ion chromatography recorded spectra at different potentials. (b) Chronoamperometric curves at different applied potentials. (c) Corresponded NH_3 yield rate (left axis) and FE (right axis).

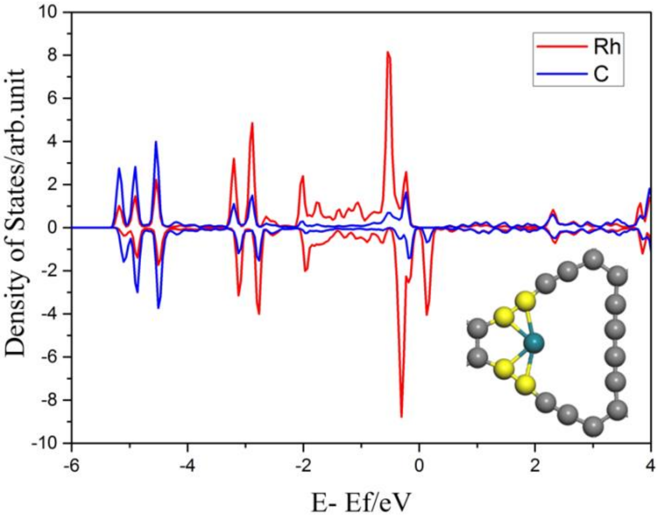

Figure S23. PDOS of Rh (green atom) and C (yellow atoms) atoms.

Table S1. Structural parameters extracted from the Rh K-edge EXAFS fitting of Rh SA/GDY. ($S0^2$ = 0.88)

Sample	Scattering pair	CN	R (Å)	σ^2 (10^{-3} Å^2)	ΔE_0 (eV)	R factor
Rh SA/GDY	Rh-C	3.92	2.1	0.09	1.16	0.00332

$S0^2$ represents the amplitude reduction factor; CN means the coordination number; R is the bond length between Rh atoms and surrounding coordination atoms; σ^2 is Debye-Waller factor; ΔE_0 is an edge-energy shift (the difference between the zero kinetic energy value of the sample and that of the theoretical model); R factor means a measure of the quality of the EXAFS fitting.

Table S2. ENRR parameters of Rh SA/GDY measured under ambient conditions.

E (V)	Integral area	Q (C)	C_{NH3} (ug mL^{-1})	FE (%)	NH_3 yield (ug h^{-1} cm^{-2})
−0.1	56446	1.43	0.09	3.25	1.37
−0.15	152330	3.85	0.27	3.60	3.97
−0.2	300862	6.62	0.53	4.12	8.01
−0.25	377360	10.47	0.67	3.27	10.10
−0.3	92020	14.71	0.16	0.54	2.33

Table S3. ENRR parameters of Rh SA/GDY tested at N_2 partial pressure of 10 atm.

E (V)	Integral area	Q (C)	C_{NH3} (ug mL^{-1})	FE (%)	NH_3 yield (ug h^{-1} cm^{-2})
–0.1	102003	2.12	0.21	4.18	3.14
–0.15	292348	4.98	0.52	5.32	7.78
–0.2	552305	9.11	0.99	5.56	14.74
–0.25	606253	13.84	1.09	4.12	16.31
–0.3	234869	23.13	0.41	0.92	6.22

Table S4. ENRR parameters of Rh SA/GDY tested at N_2 partial pressure of 30 atm.

E (V)	Integral area	Q (C)	C_{NH3} (ug mL^{-1})	FE (%)	NH_3 yield (ug h^{-1} cm^{-2})
−0.1	244469	3.62	0.43	6.1	6.48
−0.15	762550	8.04	1.37	8.71	20.56
−0.2	1362413	10.21	2.46	12.31	36.87
−0.25	775003	14.63	1.39	4.87	20.90
−0.3	359160	24.5	0.64	1.33	9.60

Table S5. ENRR parameters of Rh SA/GDY tested at N_2 partial pressure of 55 atm.

E (V)	Integral area	Q (C)	C_{NH3} (ug mL^{-1})	FE (%)	NH_3 yield (ug h^{-1} cm^{-2})
–0.1	623877	4.92	1.12	11.60	16.79
–0.15	1750609	9.12	3.16	17.71	47.43
–0.2	2732266	12.40	4.94	20.36	74.15
–0.25	1604087	19.22	2.90	7.70	43.45
–0.3	988171	35.60	1.78	2.55	26.70

Table S6. Detail parameters of Rh SA/GDY at each cycle.

Cycle	Integral area	Q (C)	C_{NH3} (ug mL^{-1})	FE (%)	NH_3 yield (ug h^{-1} cm^{-2})
1	2732266	12.4	4.94	20.36	74.15
2	2696602	12.2	4.85	20.34	72.88
3	2621484	11.91	4.74	20.26	71.11
4	2547320	11.80	4.61	19.89	69.70
5	2488337	11.61	4.50	19.81	67.40
6	2396939	11.43	4.33	19.1	65.0

Table S7. Detail parameters of the isotopic experiments under ambient conditions.

Times (h)	Q (C)	FE (%)	NH_3 yield (ug h^{-1} cm^{-2})
6	28.63	3.35	9.4
12	62.30	2.98	9.08

Table S8. ENRR parameters of Ru SA/GDY tested at N_2 partial pressure of 55 atm.

E (V)	Integral area	Q (C)	C_{NH3} (ug mL^{-1})	FE (%)	NH_3 yield (ug h^{-1} cm^{-2})
–0.1	456281	6.41	0.82	6.50	12.24
–0.15	1213320	10.71	2.19	10.43	32.82
–0.2	1745896	13.70	3.15	11.76	47.30
–0.25	1386766	18.42	2.50	6.94	37.54
–0.3	664552	31.82	1.19	1.92	17.90

Table S9. ENRR parameters of Co SA/GDY tested at N_2 partial pressure of 55 atm.

E (V)	Integral area	Q (C)	C_{NH3} (ug mL^{-1})	FE (%)	NH_3 yield (ug h^{-1} cm^{-2})
–0.2	322111	6.03	0.57	4.85	8.60
–0.25	1005734	11.29	1.81	8.20	27.18
–0.3	1237000	15.82	2.23	7.20	33.46
–0.35	858379	20.62	1.54	3.83	23.17
–0.4	456281	31.11	0.82	1.34	12.24

Table S10. ENRR performance comparison of Rh SA/GDY with other previously reported catalysts

Catalysts	Conditions	Electrolyte	NH_3 yield rate (μg h^{-1} cm^{-2})	FE (%)	Quantification method	Ref
**Rh SA/GDY**	**55 atm, 25 ℃**	**K_2SO_4 and H_2SO_4**	**74.15**	**20.36**	**Ion chromatography and ^{1}H NMR**	**This work**
**Rh SA/GDY**	**Ambient**	**K_2SO_4 and H_2SO_4**	**10.10**	**3.97.**	**Ion chromatography and ^{1}H NMR**	**This work**
Fe/Ru (Haber-Bosch method)	200~300 atm, 350~550 ℃	\	~20% (conversion rate)	\	\	[8]
Fe_3Mo_3C/C	7 atm, 25 ℃	KOH	14.74	13.55	Indophenol blue method	[9]
Ru/C	1 atm, 90 ℃	KOH	1.3	0.24	Ion chromatography	[10]
DR MoS_2	Ambient	Na_2SO_4	11.92	8.34	Indophenol blue method	[11]
MoS_2/BCCF	Ambient	Li_2SO_4	26.8	9.81	Indophenol blue method	[12]
Ru/2H-MoS_2	1 atm, 55 ℃	HCl	6.98	17.6	Indophenol blue method	[13]
Rh NF	Ambient	KOH	7.34	0.22	Indophenol blue method	[14]
FeS@MoS_2/CFC	Ambient	Na_2SO_4	8.45	2.96	Indophenol blue method	[15]
Au/TiO_2	Ambient	HCl	21.42	8.11	Indophenol blue method	[16]
Au/CoO_x	Ambient	H_2SO_4	15.1	19	Indophenol blue method and ammonia ion-selective electrode	[17]
AuSAs-NDPCs	Ambient	HCl	2.32	12.3	Indophenol blue method	[18]
Au/CeO_x-RGO	Ambient	HCl	2.21	10.1	Indophenol blue	[19]

					method	
Ru SAs/N-C	Ambient	H_2SO_4	30.84	29.6	Indophenol blue method	[20]
FL-BP NSs	Ambient	HCl	6.27	5.07	Indophenol blue method	[21]
PEBCD/C	Ambient	Li_2SO_4	2.01	2.91	Nessler's reagent	[22]
B_4C	Ambient	HCl	2.66	15.95	Indophenol blue method	[23]
$Bi_4V_2O_{11}$	Ambient	HCl	46.42	10.16	Indophenol blue method	[24]
CoS_2/NC-G	Ambient	H_2SO_4	10.00	25.9	Indophenol blue method	[25]
$BiVO_4$	Ambient	Na_2SO_4	8.60	10.04	Indophenol blue method	[26]

SI References

1. G. Li *et al.*, Architecture of graphdiyne nanoscale films. *Chem. Commun.* 46, 3256-3258 (2010).
2. J. P. Perdew, K. Burke, M. Ernzerhof, Generalized gradient approximation made simple. *Phys. Rev. Lett.* 77, 3865-3868 (1996).
3. P. E. Blochl, Projector augmented-wave method. *Phys. Rev. B* 50, 17953-17979 (1994).
4. H. J. Monkhorst, J. D. Pack, Special points for brillouin-zone integrations. *Phys. Rev. B* 13, 5188-5192 (1976).
5. S. Grimme, S. Ehrlich, L. Goerigk, Effect of the damping function in dispersion corrected density functional theory. *J. Comput. Chem.* 32, 1456-1465 (2011).
6. J. K. Norskov *et al.*, Origin of the overpotential for oxygen reduction at a fuel-cell cathode. *J. Phys. Chem. B* 108, 17886-17892 (2004).
7. A. A. Peterson, F. Abild-Pedersen, F. Studt, J. Rossmeisl, J. K. Norskov, How copper catalyzes the electroreduction of carbon dioxide into hydrocarbon fuels. *Energy Environ. Sci.* 3, 1311-1315 (2010).
8. C. J. M. van der Ham, M. T. M. Koper, D. G. H. Hetterscheid, Challenges in reduction of dinitrogen by proton and electron transfer. *Chem. Soc. Rev.* 43, 5183-5191 (2014).
9. H. Cheng, P. Cui, F. Wang, L.-X. Ding, H. Wang, High efficiency electrochemical nitrogen fixation achieved with a lower pressure reaction system by changing the chemical equilibrium. *Angew. Chem., Int. Ed.* 58, 15541-15547 (2019).
10. V. Kordali, G. Kyriacou, C. Lambrou, Electrochemical synthesis of ammonia at atmospheric pressure and low temperature in a solid polymer electrolyte cell. *Chem. Commun.* 1673-1674 (2000).
11. X. Li *et al.*, Boosted electrocatalytic N_2 reduction to NH_3 by defect-rich MoS_2 nanoflower. *Adv. Energy Mater.* 8, 1801357 (2018).
12. Y. Liu *et al.*, Dramatically enhanced ambient ammonia electrosynthesis performance by in-operando created Li-S interactions on MoS_2 electrocatalyst. *Advanced Energy Mater.* 9, 180393 (2019).
13. B. H. R. Surrnto *et al.*, MoS_2 polymorphic engineering enhances selectivity in the electrochemical reduction of nitrogen to ammonia. *ACS Energy Lett.* 4, 430-435 (2019).
14. H.-M. Liu *et al.*, Surfactant-free atomically ultrathin rhodium nanosheet nanoassemblies for efficient nitrogen electroreduction. *J. Mater. Chem. A* 6, 3211-3217 (2018).
15. Y. Guo *et al.*, Boosting nitrogen reduction reaction by bio-inspired FeMoS containing hybrid electrocatalyst over a wide pH range. *Nano Energy* 62, 282-288 (2019).
16. M.-M. Shi *et al.*, Au sub-nanoclusters on TiO_2 toward highly efficient and selective electrocatalyst for N_2 conversion to NH_3 at ambient conditions. *Adv. Mater.* 29, 1606550 (2017).
17. J. Zheng *et al.*, Tuning the electron localization of gold enables the control of nitrogen-to-ammonia fixation. *Angew. Chem., Int. Ed.* 58, 18604-18609 (2019).
18. Q. Qin, T. Heil, M. Antonietti, M. Oschatz, Single-site gold catalysts on hierarchical N-doped porous noble carbon for enhanced electrochemical reduction of nitrogen. *Small Methods* 2, 1800202 (2018).
19. S.-J. Li *et al.*, Amorphizing of au nanoparticles by CeO_x-RGO hybrid support towards highly efficient electrocatalyst for N_2 reduction under ambient conditions. *Adv. Mater.* 29, 1700001 (2017).
20. Z. Geng *et al.*, Achieving a record-high yield rate of 120.9 $\mu g_{NH3}\ mg_{cat.}^{-1}\ h^{-1}$ for N_2 electrochemical reduction over Ru single-atom catalysts. *Adv. Mater.* 30, 1803498 (2018).
21. L. Zhang, L.-X. Ding, G.-F. Chen, X. Yang, H. Wang, Ammonia synthesis under ambient conditions: Selective electroreduction of dinitrogen to ammonia on black phosphorus nanosheets. *Angew. Chem., Int. Ed.* 58, 2612-2616 (2019).
22. G.-F. Chen *et al.*, Ammonia electrosynthesis with high selectivity under ambient conditions via a Li^+ incorporation strategy. *J. Am. Chem. Soc.* 139, 9771-9774 (2017).
23. W. Qiu *et al.*, High-performance artificial nitrogen fixation at ambient conditions using a metal-free electrocatalyst. *Nat. Commun.* 9, 3485 (2018).

24. C. Lv *et al.*, An amorphous noble-metal-free electrocatalyst that enables nitrogen fixation under ambient conditions. *Angew. Chem., Int. Ed.* 57, 6073-6076 (2018).
25. P. Chen *et al.*, Interfacial engineering of cobalt sulfide/graphene hybrids for highly efficient ammonia electrosynthesis. *Proc. Natl. Acad. Sci. USA* 116, 6635-6640 (2019).
26. J.-X. Yao *et al.*, Tailoring oxygen vacancies of $BiVO_4$ toward highly efficient noble-metal-free electrocatalyst for artificial N_2 fixation under ambient conditions. *Small Methods* 3, 1800333 (2019).
